# "PP2C7s", Genes Most Highly Elaborated in Photosynthetic Organisms, Reveal the Bacterial Origin and Stepwise Evolution of PPM/PP2C Protein Phosphatases

**DOI:** 10.1371/journal.pone.0132863

**Published:** 2015-08-04

**Authors:** David Kerk, Dylan Silver, R. Glen Uhrig, Greg B. G. Moorhead

**Affiliations:** Department of Biological Sciences, University of Calgary, Calgary, Alberta, Canada; Universidade Federal de Vicosa, BRAZIL

## Abstract

Mg^+2^/Mn^+2^-dependent type 2C protein phosphatases (PP2Cs) are ubiquitous in eukaryotes, mediating diverse cellular signaling processes through metal ion catalyzed dephosphorylation of target proteins. We have identified a distinct PP2C sequence class (“PP2C7s”) which is nearly universally distributed in Eukaryotes, and therefore apparently ancient. PP2C7s are by far most prominent and diverse in plants and green algae. Combining phylogenetic analysis, subcellular localization predictions, and a distillation of publically available gene expression data, we have traced the evolutionary trajectory of this gene family in photosynthetic eukaryotes, demonstrating two major sequence assemblages featuring a succession of increasingly derived sub-clades. These display predominant expression moving from an ancestral pattern in photosynthetic tissues toward non-photosynthetic, specialized and reproductive structures. Gene co-expression network composition strongly suggests a shifting pattern of PP2C7 gene functions, including possible regulation of starch metabolism for one homologue set in Arabidopsis and rice. Distinct plant PP2C7 sub-clades demonstrate novel amino terminal protein sequences upon motif analysis, consistent with a shifting pattern of regulation of protein function. More broadly, neither the major events in PP2C sequence evolution, nor the origin of the diversity of metal binding characteristics currently observed in different PP2C lineages, are clearly understood. Identification of the PP2C7 sequence clade has allowed us to provide a better understanding of both of these issues. Phylogenetic analysis and sequence comparisons using Hidden Markov Models strongly suggest that PP2Cs originated in Bacteria (Group II PP2C sequences), entered Eukaryotes through the ancestral mitochondrial endosymbiosis, elaborated in Eukaryotes, then re-entered Bacteria through an inter-domain gene transfer, ultimately producing bacterial Group I PP2C sequences. A key evolutionary event, occurring first in ancient Eukaryotes, was the acquisition of a conserved aspartate in classic Motif 5. This has been inherited subsequently by PP2C7s, eukaryotic PP2Cs and bacterial Group I PP2Cs, where it is crucial to the formation of a third metal binding pocket, and catalysis.

## Introduction

Protein phosphorylation is an ancient covalent modification to control the biological function of proteins [1]. The prevalence of phosphorylation in biological systems was highlighted in recent mass spectrometry studies that suggested up to 70% of human proteins are phosphorylated on serine, threonine or tyrosine residues [2]. The major eukaryotic serine and threonine dephosphorylating enzymes belong to the phosphoprotein phosphatase (PPP; includes PP1, PP2A, PP3 (PP2B), PP4-PP7) and protein phosphatase Mg^2+^/Mn^2+^-dependent (PPM/PP2C) families. Both PPPs and PP2Cs form similar structural folds, however these groups have divergent primary sequences and the PP2Cs require additional metal ions for activity *in vitro* [3]. Unlike PPP enzymes that associate with additional protein regulatory subunits to confer specificity of function [4], the PP2Cs lack such regulatory subunits. Instead, almost all PP2C genes have evolved with additional domains or modules, linked to the catalytic phosphatase domain, which confer unique roles and regulation [5]. PP2Cs regulate proteins involved in fundamental signaling pathways including DNA damage repair [6], mammalian energy and glycogen metabolism [7], stress response [8], bacterial carbon/nitrogen sensing [9] and the abscisic acid hormonal response in plants [10].

PP2C enzymes were originally defined as two isoforms that had Mg^2+^-dependent activity after purification from skeletal muscle [11]. The initial solved structure of a PP2C protein, that of human PP2Cα, contained two bound Mn^+2^ ions coordinated by sets of conserved aspartate residues, leading to a classic model of protein catalysis via activated water molecules [3]. More recent results have demonstrated binding of three metal ions in structures of solved PP2Cs from both Bacteria and Eukaryotes [12–16], and the importance of additional conserved aspartate residues to both third metal binding and catalysis [17, 18]. A comprehensive picture of the evolution of PP2C metal binding capacity is still lacking.

PP2Cs are ubiquitous enzymes amongst Eukaryotes and exist as multiple genes in most organisms including 20 in humans [19], 7 in *Saccharomyces cerevisiae* [8], and a large expansion in plants, with 80 and 90 genes in *Arabidopsis thaliana* and *Oryza sativa*, respectively [19–25]. Eukaryotic PP2Cs are known to reside in the cytosol and nucleus, and proteomics studies identified these proteins in isolated mitochondria [26–28]. The functional diversity of the broad PP2C expansion in plants has thus far received limited attention, with reports focusing on the characteristics of a few scattered sub-clades [25, 29]. PP2Cs have been found in a limited number of bacterial and archaeal genomes, leading to the hypothesis that the PPM family arose in Eukaryotes and then emigrated to prokaryotes by horizontal gene transfer [30, 31]. For further details about PP2Cs the interested reader is referred to the following references [8, 25, 32–34].

We have delineated a novel eukaryotic PP2C gene lineage (“PP2C7s”) that is nearly universally distributed in Eukaryotes and therefore ancient, and which is most highly elaborated in photosynthetic Eukaryotes. Phylogenetic analysis, subcellular localization predictions, and various gene expression data demonstrate increasingly divergent sub-clades, with altered expression patterns and possible functions. Deeper phylogenetic analysis demonstrates that PP2C7s have apparently arisen from a bacterial ancestor, which likely entered eukaryotes through the ancient mitochondrial endosymbiosis event. Furthermore, these data also provide a foundation for interpreting the evolution of the catalytic mechanism of PP2Cs as a stepwise acquisition of three metal binding pockets. This architecture is shared by the PP2C7s, eukaryotic PP2Cs, and a class of bacterial PP2Cs which appear to have arisen by lateral gene transfer from Eukaryotes.

## Materials and Methods

### Candidate Sequence Search, Retrieval and Validation

Candidate PP2C7 sequences were identified using BLASTP searches [35] at NCBI (http://blast.ncbi.nlm.nih.gov/Blast.cgi), UniProt (http://www.uniprot.org/), Phytozome (http://www.phytozome.net/) or the JGI Genome Portal (http://genome.jgi.doe.gov/) with PP2C7 sequences from *Homo sapiens* (UniProtKB Accession: Q8NI37 (PPTC7_HUMAN)) or the Arabidopsis PP2C7 homologue At4g16580.1 (TAIR, http://www.arabidopsis.org/). These were then used to generate initial multiple sequence alignments, as described below. Hidden Markov Models (HMMs) were generated, and searches of databases from completely sequenced eukaryotic and prokaryotic organisms were performed as previously described [36]. Candidate sequences obtained in this fashion were supplemented further with BLASTP and HMM searches of databases of non-sequenced genomes, to identify closely related homologues (~E<1e-30). For those retrieved database PP2C7 sequences which were fragments lacking an amino terminus, attempts were made to extend them by a combination of manual searching of genome browser nucleotide sequence for unannotated ORFs, TBLASTN searches of genomic DNA with intact homologue sequences, and de novo exon predictions with GENSCAN ([37]; http://genes.mit.edu/GENSCAN.html). All candidate PP2C7 sequences were confirmed through multiple sequence alignment and phylogenetic tree inference. All sequences found are located in Table A in S1 File.

### Multiple Sequence Alignments

**Fig 1:**
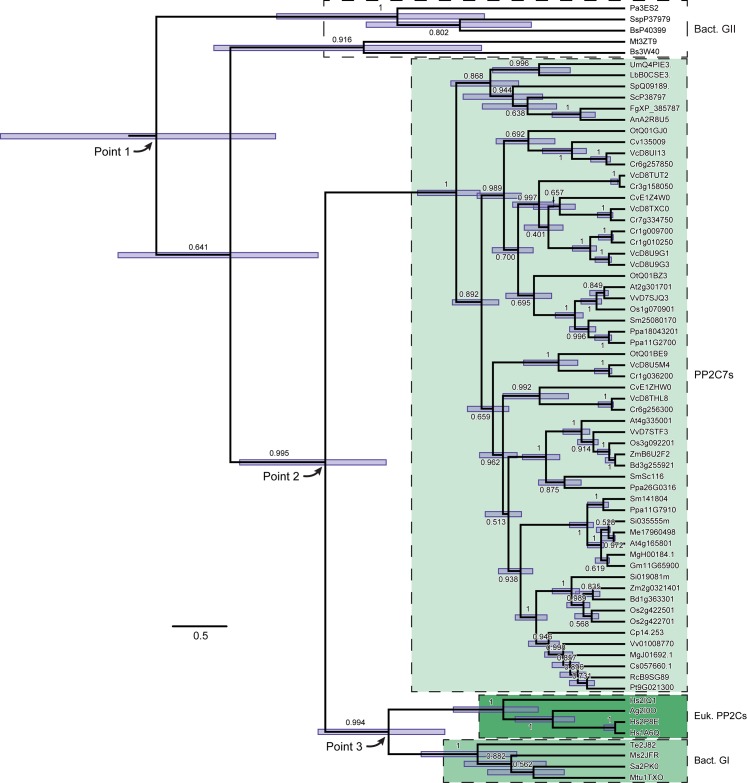
Phylogenetic orthogonal tree depicting major divergence points in the evolutionary history of modern PP2C sequences. A rooted phylogenetic tree was inferred using BEAST analysis, as detailed in “Materials and Methods”. Two independent chains were run from the same input file for 50 million cycles, collecting 10,000 trees each from the posterior distribution. A “burn-in” of 1,000 trees was discarded from each sample and the remaining trees pooled manually. The log files for the two runs were combined, and are available as supporting information (S4 File). In this figure the root is located in the upper left of the image. Depicted is the maximum clade credibility tree from the posterior distribution tree sample. Nodes display the 95% high posterior density interval in blue. Each branch is labeled with the posterior probability (max = 1.0). Point 1, Point 2 and Point 3 are discussed in the text. This tree is based on the amino acid sequence alignment presented in Fig A in S2 File, Panel 1.

**Fig 2:**
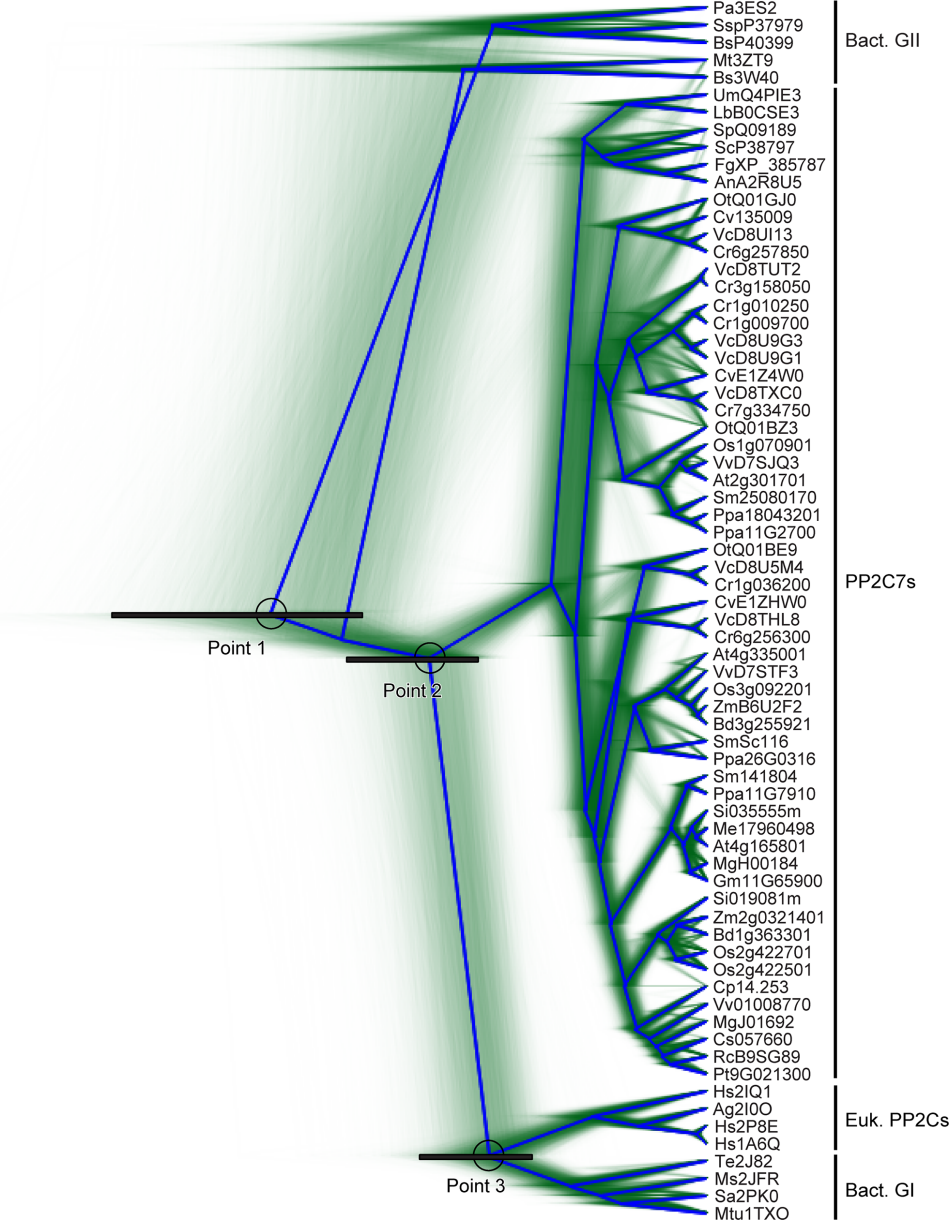
Topological uncertainty in the phylogenetic tree summarizing PP2C sequence evolutionary history. This tree is an alternate display of the same BEAST analysis data used to generate Fig 1. Green lines represent traces of individual trees from amongst the posterior distribution tree sample. In blue is the consensus tree with the highest clade support (“root canal”). Points 1, 2, and 3 are discussed in the text. Each represents a node with a black bar indicating the 95% high posterior density interval.

**Fig 3:**
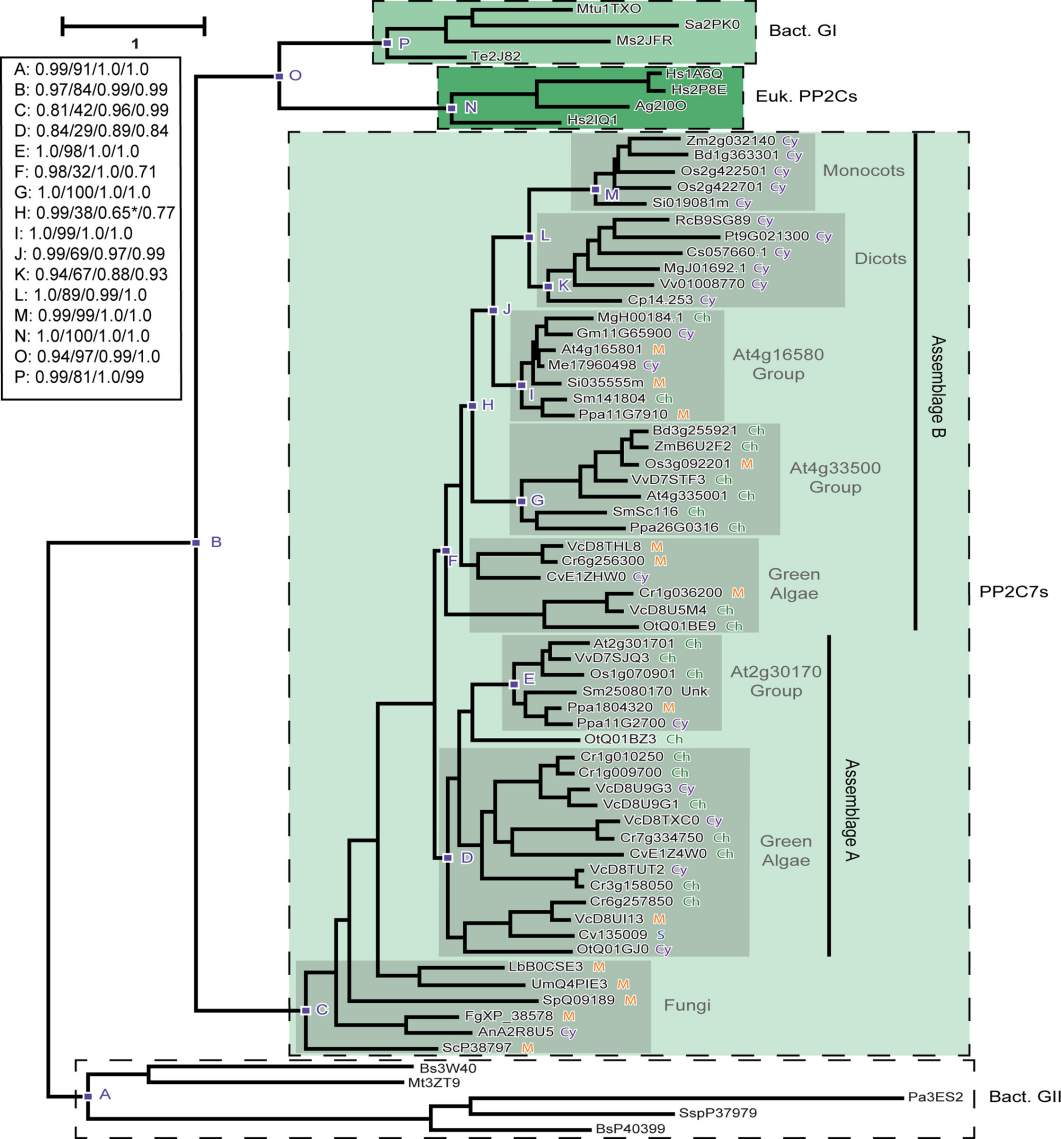
Phylogenetic orthogonal tree depicting interrelationships between representative PP2C7 sequences from plants, green algae, and fungi. Inference of unrooted phylogenetic trees was performed as outlined in “Materials and Methods.” A typical example is shown. The most crucial nodes are labeled. Node support values with the four inference methods (PhyML [aBayes], RAxML [RBS], MrBayes [PP], PhyloBayes_MPI [PP]) are tabulated in the Figure, separated by slashes (“/”). Support values for all trees are summarized in Table N in S1 File. Predicted in silico subcellular localizations are represented as follows: Ch, chloroplast; Cy, cytosol; M, mitochondria; S, signal peptide; Unk (unknown), sequence fragment lacking native amino terminus. Sequences used in phylogenetic tree generation are listed in Table A in S1 File, while compiled in silico subcellular localization data can be found in Table B in S1 File (non-photosynthetic organisms) and Table F in S1 File (photosynthetic organisms). * = Three algal sequences included in this cluster.

**Fig 4:**
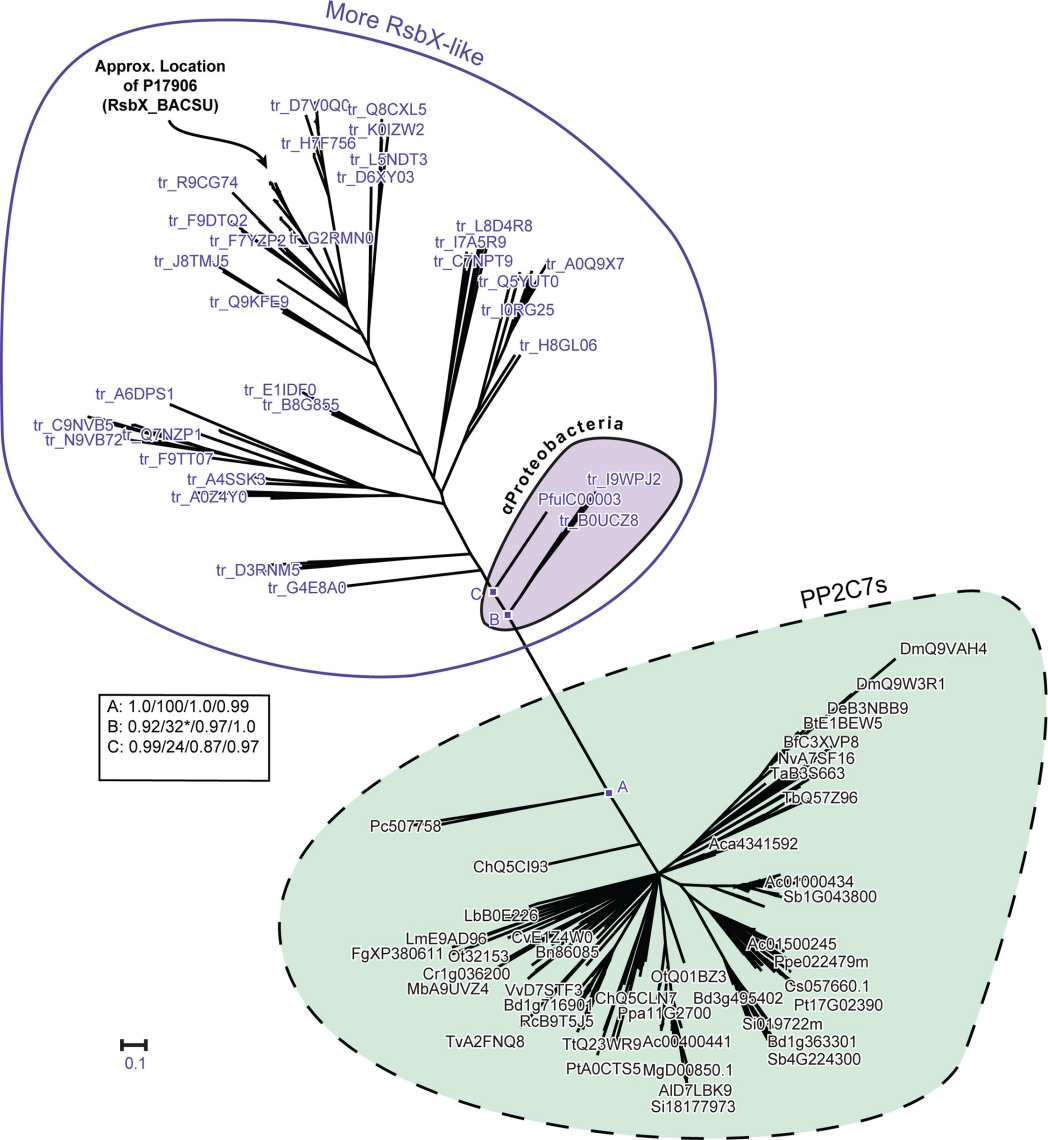
Phylogenetic radial tree depicting interrelationships between PP2C7 sequences and bacterial Group II PP2C sequences. The PP2C7 set is a very large and diverse one (238 sequences) and the bacterial Group II sequences are of the “GN” type, from the “More RsbX-Like” assemblage (144 sequences) (sequence varieties described in the text). For this analysis the sequences of the Myxococcales group have been removed (A9GSF9, A6FYN9, A9GWA1, E3FWN8, L7U8R0) (see text for rationale). Inference of unrooted phylogenetic trees was performed as outlined in “Materials and Methods.” The most crucial nodes are labeled. Node support values with the four inference methods (PhyML [aBayes], RAxML [RBS], MrBayes [PP], and PhyloBayes_MPI [PP]) are tabulated in the Figure, separated by slashes (“/”). Support values for all trees are summarized in Table N in S1 File. Preliminary analyses showed that attainment of consistent tree topologies between the different inference methods required removal of the following sequences: Q2RIF7, H1Z3D3, C9R9C1, B1I2G2, G2MXY4, Q8RAY2, E4Q3X2. The cluster of sequences from α-Proteobacteria is indicated. The approximate location in the tree of the reference sequence BsP17906 (RsbX_BACSU) is indicated. This tree is based on the amino acid sequence alignment presented in S1(B) Fig. * = αProteobacteria cluster separated into adjacent fragments in this tree.

**Fig 5:**
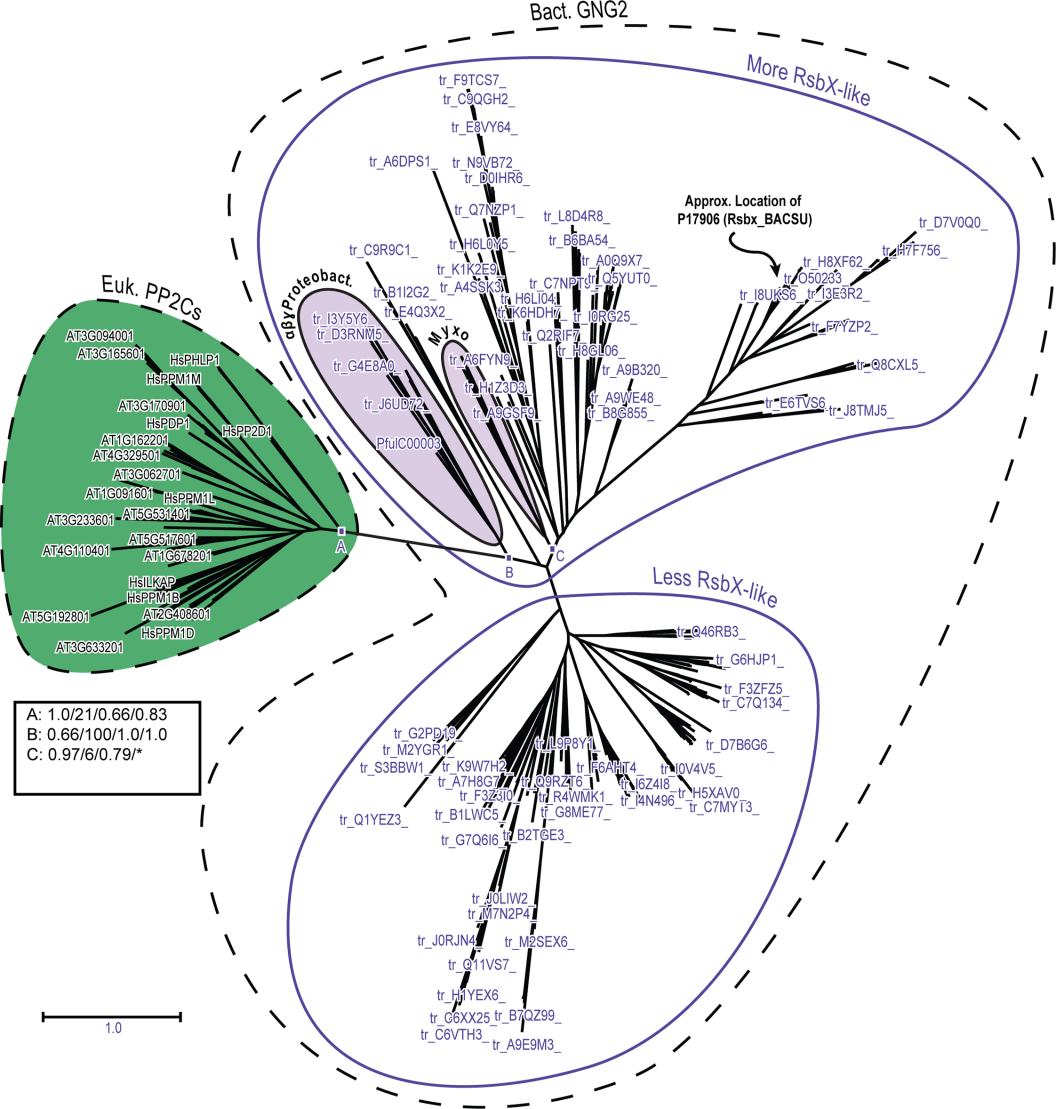
Phylogenetic radial tree depicting interrelationships between eukaryotic PP2C sequences and bacterial Group II PP2C sequences. The eukaryotic PP2C set consists of the combined sequences (excluding PP2C7s) from Arabidopsis and human (96 sequences total—HsTAB1 excluded). The bacterial Group II sequences are of the “GN” type, including the “More RsbX-Like” and “Less RsbX-Like” assemblages (328 sequences total) (see text for explanation of sequence varieties). Inference of unrooted phylogenetic trees was performed as outlined in “Materials and Methods.” The most crucial nodes are labeled. Node support values with the four inference methods (PhyML [aBayes], RAxML [RBS], MrBayes [PP], and PhyloBayes_MPI [PP]) are tabulated in the Figure, separated by slashes (“/”). Support values for all trees are summarized in Table N in S1 File. The cluster of sequences from αβγ-Proteobacteria is indicated. “Myxo” designates sequences from the Myxococcales (δ-Proteobacteria). The approximate location in the tree of the reference sequence BsP17906 (RsbX_BACSU) is indicated. This tree is based on the amino acid sequence alignment presented in Fig A in S2 File, Panel 3. * = Myxococcales unresolved from other sequences in this tree.

**Fig 6:**
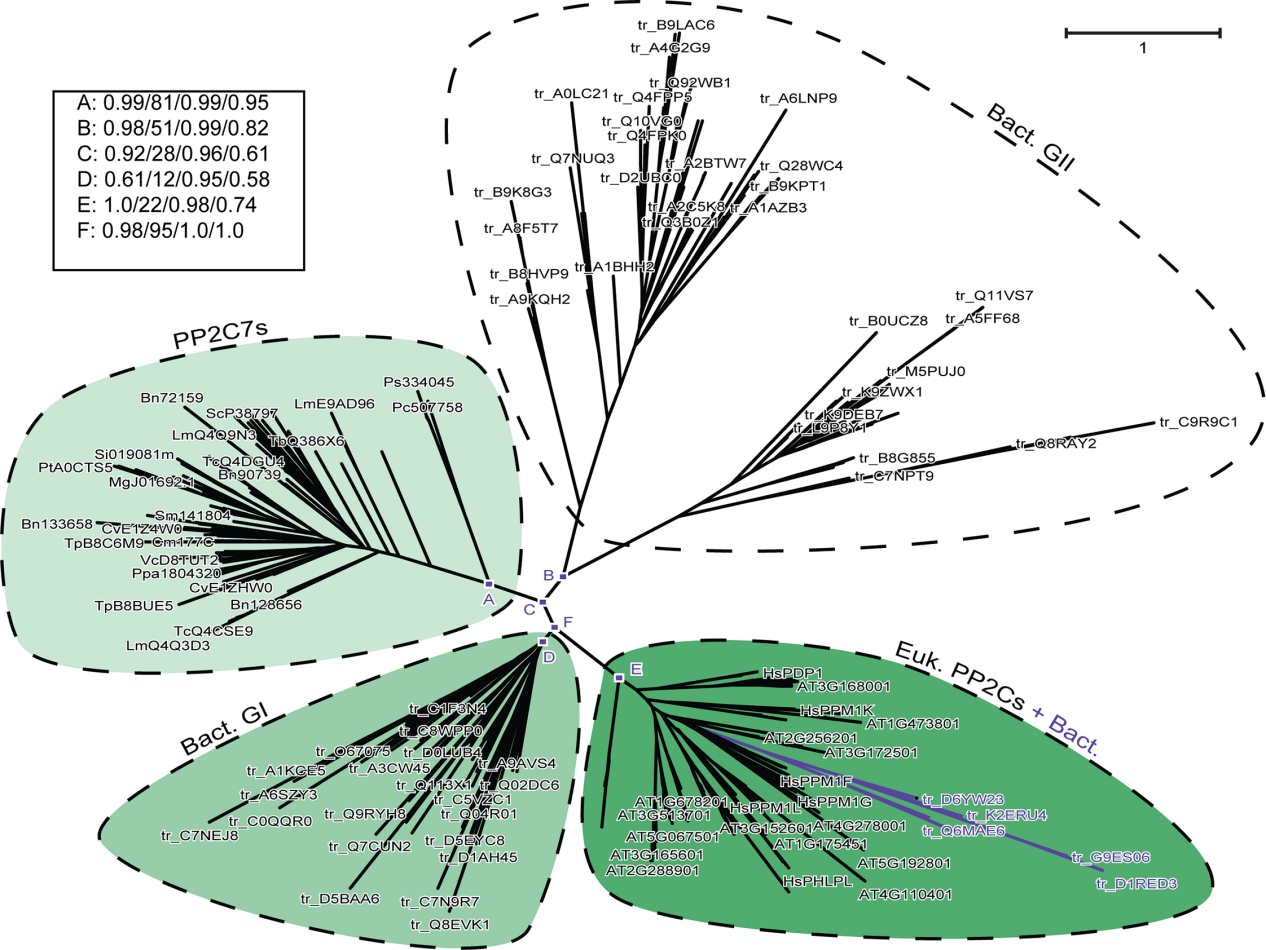
Phylogenetic radial tree depicting a large scale comparison between PP2C7 sequences, bacterial Group II sequences, bacterial Group I sequences and eukaryotic PP2C sequences. There are 102 representative PP2C7 sequences, and 49 representative bacterial Group I sequences. The eukaryotic PP2C set consists of the combined sequences (excluding PP2C7s) from Arabidopsis and human (96 sequences total—HsTAB1 excluded). The bacterial Group II sequences include representatives from both “Bulk” (50 sequences) and “GN” types (38 sequences) (see text for explanation of sequence varieties). There are also 9 “Eukaryotic-Like” bacterial PP2C sequences (see text for explanation). Inference of unrooted phylogenetic trees was performed as outlined in “Materials and Methods.” The most crucial nodes are labeled. Node support values with the four inference methods (PhyML [aBayes], RAxML [RBS], MrBayes [PP], and PhyloBayes_MPI [PP]) are tabulated in the Figure, separated by slashes (“/”). Support values for all trees are summarized in Table N in S1 File. The cluster of “Eukaryotic-Like” bacterial PP2C sequences within the eukaryotic PP2C clade is indicated by colored lines. This tree is based on the amino acid sequence alignment presented in Fig A in S2 File, Panel 4.

**Fig 7:**
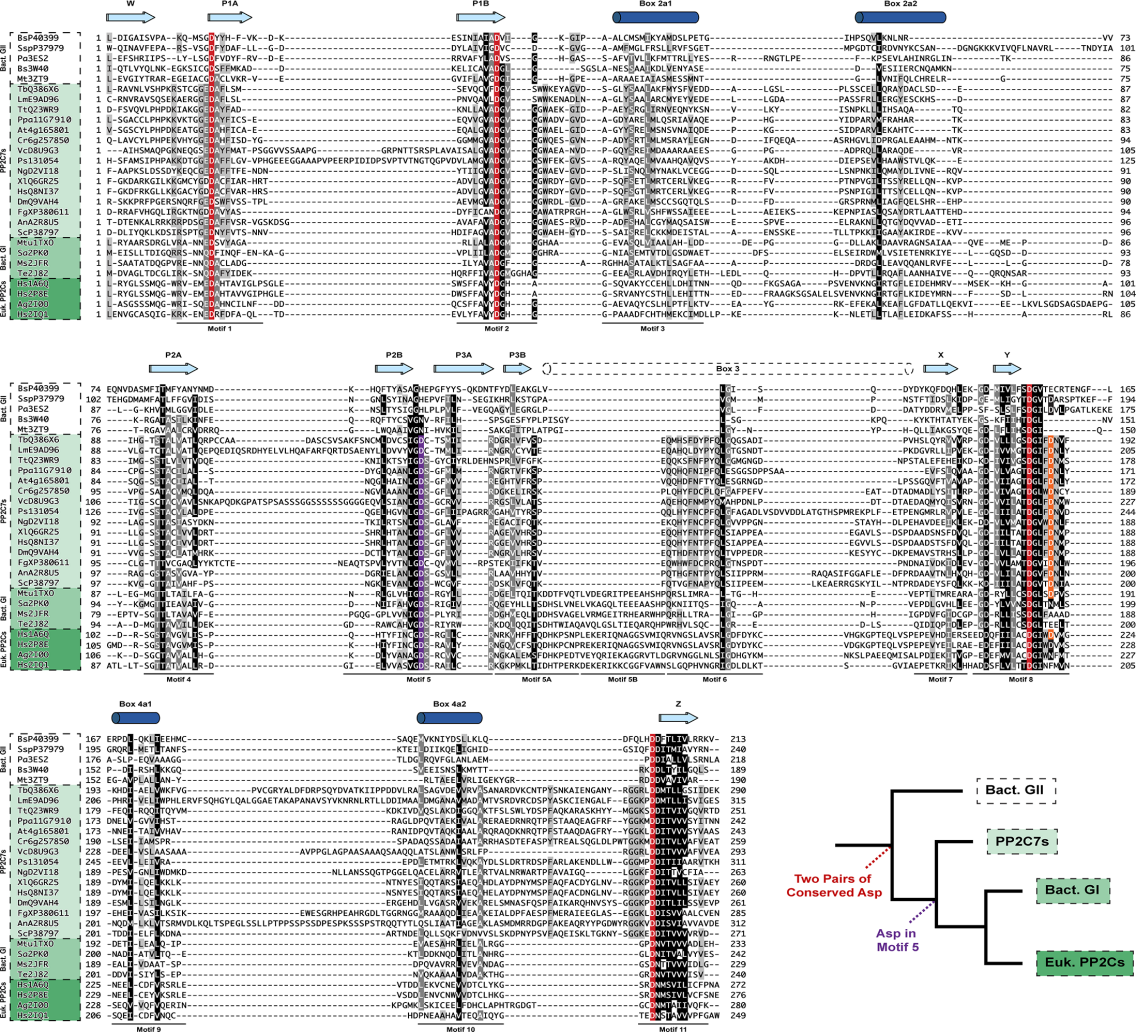
Structure-guided alignment of bacterial Group II, PP2C7, bacterial Group I, and eukaryotic PP2C sequences. Information from solved structures of bacterial Group II, bacterial Group I, and eukaryotic PP2Cs (indicated by their four-character PDB codes) was used to guide this alignment, as detailed in “Materials and Methods”. Above the sequences are shown conserved beta-strand and α-helical secondary structure elements. “Box” refers to a more variable region in multiple solved structures. For a secondary structure diagram, including element numbering, see Fig F in S2 File. Sequence motifs are as given in [84]. Universally conserved aspartates involved in metal coordination are given in red. Aspartates conserved in some but not all sequences are given in purple and orange (see text for discussion). The inset shows a simplified phylogenetic tree, with the proposed evolutionary advent of critical aspartate residues indicated. See Table A in S1 File for a listing of PP2C7 sequences. Bacterial Group II sequences without solved structures are from UniProt. Species for sequences are as follows: Bs (Bacillus subtilis); Ssp (Synechocystis sp.); Pa (Pseudomonas aeruginosa); Mt (Moorella thermoacetica); Tb (Trypanosoma brucei); Lm (Leishmania major); Tt (Tetrahymena thermophila); Ppa (Physcomitrella patens); At (Arabidopsis thaliana); Cr (Chlamydomonas reinhardtii); Vc (Volvox carteri); Ps (Phytophthora sojae); Ng (Naegleria gruberi); Xl (Xenopus laevis); Hs (Homo sapiens); Dm (Drosophila melanogaster); Fg (Fusarium graminearum); An (Aspergillus niger); Sc (Saccharomyces cerevisiae); Mtu (Mycobacterium tuberculosis); Sa (Streptococcus agalactiae); Ms (Mycobacterium smegmatis); Te (Thermosynechococcus elongatus); Ag (Anopheles gambiae).

Structure-guided alignments were made from sets of PP2C sequences with solved structures using the 3DCOMB function of the RaptorX server ([38, 39]; http://raptorx.uchicago.edu/DeepAlign/submit/). Solved structures used were: ((1A6Q, 2P8E, 2I0O, 2IQ1 –eukaryotic PP2Cs); (1TXO, 2PK0, 2JFR, 2J82 –bacterial Group I); (3ES2, 3W40, 3ZT9 –bacterial Group II)). Additional sequences (eukaryotic PP2C7s) were added with the sequence to profile function of ClustalO [40, 41] at the Mobyle Portal (http://mobyle.pasteur.fr/cgi-bin/portal.py#forms::clustalO-sequence). Alignments were then visualized and hand-edited within GeneDoc ([42]; http://www.nrbsc.org/gfx/genedoc/). In some instances local sequence regions were further aligned using a regional re-alignment capability of MAFFT (Ruby script kindly supplied by Dr. Kazutaka Katoh, modified for Windows by Justin Kerk) (http://mafft.cbrc.jp/alignment/software/regionalrealignment.html) using the following parameters (treeoption—6merpair; realign—localpair—maxiterate 100—bl 45—op 3.0). Phylogenetic trees were inferred as detailed below. This procedure formed the basis for the alignment corresponding to the phylogenetic trees presented in Figs 1, 2 and 3 (Fig A in S2 File (Panel 1)), and the alignment in Fig 7. A large alignment consisting of eukaryotic PP2C7, bacterial Group II, bacterial Group I and the PP2C sets from human and *Arabidopsis* was initially constructed by MAFFT ([43]; http://mafft.cbrc.jp/alignment/server/), using the BLOSUM45 scoring matrix, and the E-INS-I option (multiple conserved domains and long gaps). Editing of this alignment within GeneDoc was guided by the previous structure-guided alignments detailed above. At some points in the editing process the above cited MAFFT regional realignment procedure was also used. This procedure formed the basis for the alignment in Fig A in S2 File (Panel 4) (corresponding to the phylogenetic tree presented as Fig 6). Finally, large sets of bacterial Group II PP2Cs (GNG2s), with either PP2C7s or eukaryotic PP2Cs, were aligned with MAFFT, using the BLOSUM45 scoring matrix with the E-INS-I option, and manually edited within GeneDoc. In order to decrease the computational load during tree inference, the bacterial Group II component of these alignments was pruned to sequences sharing 80% identity or less, using the ExPasy “Decrease Redundancy” tool (http://web.expasy.org/decrease_redundancy/). This procedure formed the basis for the alignments in Fig A of S2 File (Panels 2, 3, and 5) (corresponding to the phylogenetic trees presented as Figs 4, 5, and Fig E in S2 File.). As necessary during this multiple sequence alignment work, files were interconverted between various formats using the online format converters at either the Mobyle Portal (http://mobyle.pasteur.fr/cgi-bin/portal.py#forms::squizz_convert), or the Phylogeny.fr site (http://www.phylogeny.fr/version2_cgi/data_converter.cgi).

### Phylogenetic Tree Inference

ProtTest, version 2.4 ([44]; http://darwin.uvigo.es/software/prottest2_server.html) was used with completed multiple sequence alignments to assess the optimal amino acid substitution model to use for subsequent phylogenetic tree inference. In all instances, the Le and Gascuel (LG) substitution model [45] with Gamma-distributed between sites rate variation (LG + G) was optimal, by both the AIC (Akaike Information Criterion) and BIC (Bayesian Information Criterion). Multiple sequence alignments were subjected to phylogenetic tree inference at both the CIPRES Science Gateway ([46]; http://www.phylo.org/index.php/portal/) and locally. Maximum likelihood analysis (RAxML version 8.1.11; [47]; http://www.exelixis-lab.org/) producing unrooted trees was run at CIPRES under the LG substitution model, four discrete Gamma rate categories (LG + G4), using a maximum of 1,000 rapid bootstraps or until automatic convergence was reached. Bayesian analysis (MrBayes version 3.2.3; [48]; http://mrbayes.sourceforge.net/) producing unrooted trees was performed at CIPRES, using two runs with four independent chains, under the Le and Gascuel (LG) substitution model [45], with four discrete Gamma rate categories (LG + G4), running to a maximum of 40 million tree generations or until automatic convergence (average SD of split frequencies < 0.010) was achieved. Bayesian analysis (PhyloBayes_MPI version 1.3b; [49]; http://megasun.bch.umontreal.ca/People/lartillot/www/downloadmpi.html) producing unrooted trees was run on the WestGrid system of Compute Canada (https://computecanada.ca/index.php/en/), using two independent chains, under the LG substitution model, with four discrete Gamma rate categories (LG + G4). Rooted trees were produced by BEAST (version 1.8.1) [50] analysis at CIPRES, using two independent chains, under the LG substitution model with four discrete Gamma rate categories (LG + G4), running a maximum of 50 million cycles. A lognormal relaxed clock (uncorrelated) was used for data reported herein (ucld.stdev = 0.335, coefficient of variation = 0.336). For the Bayesian methods, which proceed by Markov Chain Monte Carlo (MCMC) sampling of the posterior distribution, a portion of the tree samples collected was discarded as a “burn-in” (MrBayes 25%, BEAST 10%, PhyloBayes_MPI 20%). Maximum likelihood analysis (PhyML-aBayes version 3.0.1beta; [51]; http://www.atgc-montpellier.fr/phyml/versions.php) producing unrooted trees was run locally, under the LG model, with four discrete Gamma rate categories (LG + G4), with all other parameters at defaults, through 25 random starts, employing an initial parsimony input tree, and subtree pruning and regrafting (SPR) moves. Figs 1 and 2 present rooted trees from BEAST analyses, with support values given on branches in Fig 1. Other trees are unrooted and represent a typical topology observed in the other four inference methods, with support produced by each method given at the most critical nodes. The support values for all the trees are summarized in Table N in S1 File. For Bayesian methods (MrBayes, PhyloBayes_MPI, BEAST), support represents the posterior probability (PP; maximum value = 1.00). For the PhyML maximum likelihood method, support represents a Bayesian-like transformation of the approximate likelihood ratio test value (aBayes [51]; [maximum value = 1.00]). For the RAxML maximum likelihood method, support represents rapid bootstrap support (RBS; [maximum value = 100]). Primary tree files for all inference methods are provided as Supporting Information (S3 File).

### Subcellular Localization Prediction

Subcellular localization predictions were generated using the methods given previously in [36]. A sequence was judged to have a mTP (mitochondrial targeting peptide) (“M”) by majority rule consensus of the set of predictions, provided a minimum of four techniques supported this prediction (i.e. 4/9 for non-plant species). This criterion was chosen because the mouse PP2C7 sequence (UniProtKB Accession: Q6NVE9 (PPTC7_MOUSE)) is known to be mitochondrial by experiment [28], and also achieved a consensus of 4/9 positive predictions in our tests. Most of the techniques have their own internal thresholds for compartment predictions, and automatically convert the sequence score into a compartment prediction. However MITOPROT simply reports a probability of mitochondrial localization. We utilized a threshold with this technique of ≥ 0.700 since the mouse PP2C7 sequence scored 0.7012. In a similar fashion to the consensus criterion for mitochondrial localization, we deemed sequences to be chloroplastic (“Ch”) if they achieved a majority of predictions for this compartment totalling four or more of the battery of ten methods. Sequences which obtained four or more predictions for a signal peptide were designated as “S” (endomembrane system localized or secreted). Sequences which failed to obtain a majority consensus prediction for any localization signal (mTP, cTP (chloroplast transit peptide), SP (signal peptide)) were deemed to be cytoplasmic (“Cy”). For green algal sequences, the recently described PredAlgo algorithm [52] has been shown to correct the tendency for previous prediction methods to over-represent mitochondrial predictions and under-represent chloroplast predictions. Therefore if there was disagreement between the consensus prediction and that of PredAlgo, the latter prediction was used.

### Analysis of Accessory Sequence Domains and Motifs

Sequences confirmed as PP2C7s were searched for previously-characterized accessory domains using the batch mode at NCBI CDD ([53]; http://www.ncbi.nlm.nih.gov/Structure/bwrpsb/bwrpsb.cgi). Domain hits other than PP2C models were further investigated if E < 0.01. Individual sequences were also analyzed for secondary domains using HHPred ([54]; http://toolkit.tuebingen.mpg.de/hhpred) using as targets either solved PDB structures, or sequence models (CDD, Pfam, PANTHER, SMART). Coiled-coil domains were identified using PCOILS ([55]; http://toolkit.tuebingen.mpg.de/pcoils). Additional sequences having defined domain architectures were identified using NCBI CDART (Conserved Domain Architecture Retrieval Tool) ([56]; http://www.ncbi.nlm.nih.gov/Structure/lexington/lexington.cgi). In some instances the sequences to homologues of multi-domain PP2C7 proteins were obtained by TBLASTN [57] searching of the NCBI WGS (whole genome shotgun) sequence database.

Amino-terminal sequences of plant PP2C7 proteins were obtained by removal of the phosphatase domain defined by sequence alignments and any carboxy-terminal residues. Novel motifs in these plant PP2C7 amino-terminal sequences were inferred using GLAM2 (Gapped Local Alignment of Motifs) ([58]; http://meme.nbcr.net/meme/cgi-bin/glam2.cgi), using 100 maximum aligned columns and 5,000 iterations without improvement. Motif data are presented both as sequence logos (from the original GLAM2 output) and as conventional sequence alignments (abstracted from GLAM2 text output and displayed in GeneDoc). Inferred GLAM2 motifs were used to search the NCBI non-redundant protein database using GLAM2SCAN (Scanning with Gapped Motifs) ([58]; http://meme.nbcr.net/meme/cgi-bin/glam2scan.cgi).

### HMM–HMM Comparisons

HMMs were used to summarize the properties of alignments for several sub-groups of PP2C sequences (bacterial Group II [“Bulk”, GNG2, GNG2 sub-assemblages], PP2C7s). HMMs were compared using HHAlign at the Bioinformatics Toolkit ([59]; http://toolkit.tuebingen.mpg.de/). HHPred [54] was used with individual query sequences (PP2Cs found in Bacteria) to compare them to solved PP2C structures.

### Plant Gene Expression and Co-Expression Network Data

Plant gene expression data were accessed at PlaNet ([60]; http://aranet.mpimp-golm.mpg.de/) and Genevisible/Genevestigator ([61]; http://genevisible.com/search). Additional data for maize PP2C7 genes were obtained from [62] and for rice PP2C7 genes from [23], and from RiceXPro ([63]; http://ricexpro.dna.affrc.go.jp/). Plant gene co-expression network data for Arabidopsis were accessed at ATTED-II ([64], [65]; http://atted.jp/), and for rice at RiceFREND ([66]; http://ricefrend.dna.affrc.go.jp/). Arabidopsis promoter element analysis was performed using the tools within ATTED-II. Diurnal gene expression data for Arabidopsis was accessed at the NCBI GEO (Gene Expression Omnibus) site (http://www.ncbi.nlm.nih.gov/gds/) under the title: “Gene expression and carbohydrate metabolism through the diurnal cycle” (Series GSE6174). Numerical data were then placed into Microsoft Excel, where calculations were performed and graphs constructed.

### PP2C Structural Comparisons

The secondary structures of solved PP2C sequences were visualized using the “Protein” link of the EBI PDBSum resource ([67, 68]; http://www.ebi.ac.uk/pdbsum/). The same set of solved structures was examined as listed in the above section on *Multiple Sequence Alignment*. A nomenclature system was devised that takes into account the fact that different PP2C structures have a set of conserved secondary structure elements, amidst variable regions which may include individual, non-conserved structural elements.

## Results

### "PP2C7s" are a distinct, widely distributed eukaryotic PP2C class

While accumulating a large PP2C protein sequence set from a number of eukaryotic organisms and generating multiple sequence alignments and phylogenetic trees, we noticed a distinct clade emerging which contained the protein sequences PTC7 (**p**hosphatase **t**wo **C** 7) from *Saccharomyces cerevisiae* and PPTC7 (**P**hosphatase **PTC7** homologue) from *Homo sapiens*. We followed up on these initial observations by searching the protein databases of various completely sequenced eukaryotic genomes (see “Materials and Methods”). The result was the accumulation of a set of over 250 sequences, from more than 60 species (see Table A in S1 File). The only organisms which failed to have any PP2C7 representatives were species from the genus *Giardia* (*G*. *lamblia*, *G*. *intestinalis*) and *Entamoeba histolytica*. PP2C7 sequences are found in a very broad range of eukaryotic species, comprising all major eukaryotic groups. Generally organisms possess one or a few PP2C7s–however, plant species may contain as many as 17. The finding of a distinctive group of PP2C7 sequences widespread in all major eukaryotic groups, with very few tested species lacking them, indicates that this type of gene was present early in eukaryotic evolution.

### PP2C7s have a wide variety of predicted subcellular localizations

Experimental work in *S*. *cerevisiae* (immunofluorescence microscopy) [69] and *Mus musculus* (cell fractionation and proteomics) [28] shows that in both organisms the PP2C7 protein is localized in mitochondria. We therefore used bioinformatic techniques to generate predictions for subcellular localization in our PP2C7 sequence set. A battery of tests was used (10 in all) [36, 70], and a summary of results for non-photosynthetic organisms is presented in Table B in S1 File. Consistent with the earlier experimental observations, most tested animal sequences (16 of 19) and fungal sequences (10 of 13) produced a primary prediction indicating a mitochondrial targeting peptide (mTP). Amongst protists, 20 of 36 sequences had a primary prediction for a mTP. Most other sequences had a primary cytoplasmic prediction, with the exception of one sequence from *Paramecium tetraurelia* (A0CTS5) which had a predicted signal peptide (SP). Several protist species had sets of sequences each containing members with distinct primary subcellular localization predictions, nearly always mitochondrial versus cytoplasmic (8 species). The exception again was *Paramecium tetraurelia* with a predicted mitochondrial and a predicted SP containing sequence. Sequences from photosynthetic organisms displayed an even greater diversity of predicted subcellular localizations (including many chloroplastic) and will be considered separately later in this report.

### PP2Cs have a broad distribution in Bacteria

As a prelude to investigating the interrelationships between PP2C7s and other previously documented types of PP2C sequences, we sought to update our knowledge of bacterial PP2Cs. Previous work has described two distinct sequence classes, designated Group I and Group II [71]. We searched large databases from completely sequenced Bacteria and Archaea, and in agreement with previously published results [30, 31], we found very few sequences of either type in the Archaeal genome database (~35 candidates for each type above the HMM statistical exclusion threshold (E~0.01)). In contrast, we found over 1000 bacterial Group I sequences (Table C in S1 File), and over 1500 bacterial Group II sequences (Table D in S1 File). The lists of species positive for each sequence type are presented in Table E in S1 File. These data establish that PP2C sequences of the Group I and Group II type are nearly ubiquitous in hundreds of species of bacteria, and therefore likely constitute an important point of regulation of bacterial protein phosphorylation.

### The earliest detectable point in PP2C evolutionary history lies within the Bacteria

We next sought to establish the principal divergence points in the evolutionary history of modern PP2C sequences, including their earliest detectable origin. Our method was a phylogenetic analysis of the evolutionary interrelationships between the newly described PP2C7 lineage, other eukaryotic PP2Cs, and the bacterial Group I and Group II PP2Cs. We collected a representative sequence group of each type (largest for the PP2C7s), placed them into a multiple sequence alignment guided by the structures of solved eukaryotic PP2C, bacterial Group I and bacterial Group II PP2C sequences (shown in Fig A in S2 File, Panel 1), and inferred a rooted phylogenetic tree with BEAST analysis (see “Materials and Methods” for details). The result is shown in Fig 1. This is the tree with maximal support drawn from the large posterior distribution tree sample (the maximum clade credibility tree). The root of the tree lies in the upper left of the figure. This analysis allows a quantitative estimate of temporal uncertainty in the tree nodes. Error bars (95% high posterior density intervals) are indicated for each node. Time may be envisioned as progressing from left to right along the horizontal axis. Points 1, 2, and 3 represent candidates for important divergence points (corresponding to tree nodes) for ancestral sequences. It can be seen that there is a degree of overlap between the error bars for Point 1 and Point 2, and for Point 2 and Point 3. Hence it is not possible to designate discrete unambiguous times for each of these branching events.

In order to explore the interrelationships between these divergence points more thoroughly, we generated another display of this same BEAST analysis which highlights not only the temporal, but also the topological uncertainty in the tree. This is presented as Fig 2. The outline density in green represents traces of the topology of all the trees in the posterior distribution tree sample. In blue is the best supported consensus tree, corresponding to the tree shown in Fig 1. Points 1, 2, and 3 are presented as in Fig 1. Topological uncertainty manifests itself as reduced intensity green “phantom lines” which clearly differ from the pattern of the consensus tree in blue. Some of these are readily observable in the fine structure of the branching patterns within the PP2C7 group, and also amongst the bacterial Group II sequences–whether they should be retained as a common group or split into subgroups. However there are no such discordant line tracings detectable emerging from Points 1, 2, and 3. This indicates that despite uncertainty as to their precise dates, Points 1, 2, and 3 can be accepted as representing distinct divergence points within the history of the sequences in this analysis. It is clear that the root of the tree (which ends at the node in Point 1), representing the most ancient element in the tree, lies between bacterial groups (see also Fig 1). This is consistent with the inference that the common ancestor of all modern PP2C sequences first arose from within the Bacteria. The simplest interpretation of Point 2 and Point 3 is that each represents a eukaryotic ancestor in the subsequent history of PP2C sequence diversification.

### The greatest expansion and diversification of PP2C7s has occurred in plants

The largest contingent of PP2C7 genes and proteins was observed in photosynthetic Eukaryotes, especially plants. In this group the maximum number of PP2C7 proteins was 17 (*Populus trichocarpa*) (median = 6), compared with a maximum of 5 (*Phytophthora capsici*, *Phytophthora sojae*) in non-photosynthetic protists (median = 3), a maximum of 2 (three species) in Fungi (median = 1), and a maximum of 3 (*Drosophila melanogaster*) in animals (median = 1). In plants there are many instances of alternative protein isoforms from the same gene locus, with some loci having more than five isoforms (for example *Oryza sativa* Os3g09220 has six isoforms). There is also a great variety of predicted subcellular localizations in photosynthetic Eukaryotes. This data is summarized in Table F in S1 File. A majority of the proteins (97 of 182 [53.3%]) have primary predictions of organellar localization (chloroplasts and mitochondria), in addition there are many with primary predictions of cytoplasmic localization, and a few primary predictions of a SP.

We next sought to examine in more detail the interrelationships of various PP2C7 sequences within photosynthetic organisms. We utilized the same sequence alignment previously analyzed in Figs 1 and 2, which contains a large group of PP2C7 sequences from green algae and plants along with comparison fungal sequences. We built unrooted phylogenetic trees by the four inference methods discussed in detail in the Methods, and present a typical representative as Fig 3. The topology of this tree has two main features. First, the bacterial Group II sequences (Node A) are most closely related to the PP2C7s (Node C) (groups joined at Node B). Second, the eukaryotic PP2C sequences (Node N) are most closely related to the bacterial Group I sequences (Node P) (groups joined at Node O). These same topological relationships are evident in the trees in Figs 1 and 2. Within the PP2C7s, there are two major sequence assemblages, which we have designated “A” and “B”. Each is characterized by basal green algal sequences and more derived plant sequences. These assemblages indicate an ancient fundamental split in PP2C7 evolution within green photosynthetic eukaryotes. “Assemblage A” (Node D) contains a single plant sequence group, which includes the protein from the Arabidopsis gene locus At2g30170 (Node E). “Assemblage B” (Node F) contains four interrelated plant sequence groups. Most basal is a group which includes Arabidopsis locus At4g33500 (Node G). Next is a group which includes the Arabidopsis locus At4g16580 (Node I). Finally, there are two additional sequence groups, one from Dicot species (but lacking an Arabidopsis representative) (Node K), and another from Monocot species (Node M). Sequence group relationships are more strongly supported in the derived regions of this assemblage (Nodes J and L) than in the more basal region (Node H). In terms of previously published phylogenetic work, the Arabidopsis sequences in our analysis correspond to Clade K plus its closely associated singleton sequence (in the following reference Fig 2) [25].

Fig 3 also presents for each sequence the consensus predicted subcellular localization. It is evident from inspection of the two assemblages that they differ in the apparent frequency of chloroplastic localization: Assemblage A being higher (11/20) and Assemblage B being lower (10/31). We constructed another larger sequence alignment encompassing all identified full-length green algal and plant sequences, and derived a phylogenetic tree. The topology of this tree (data not shown) also demonstrated these two large sequence assemblages. This tree was used to order the table summarizing all photosynthetic organism subcellular localization predictions, which is presented as Table F in S1 File. Examination of the larger dataset contained there reinforces and expands on the trends outlined above. Sequence Assemblage A is predominantly chloroplastic (70.5% [31/44]), whereas Assemblage B is predominantly non-chloroplastic (74.2% [95/128]). Furthermore, there is a marked difference between the predominant localization predictions of several of the plant sequence groups. The At2g30170 group (in Assemblage A) is predominantly chloroplastic (75% [21/28]), whereas the At4g16580 and Dicot/Monocot groups (in Assemblage B) are predominantly mitochondrial (56.8% [25/44]) and cytoplasmic (Dicot: 86.2% [25/29]; Monocot: 95% [19/20]), respectively.

We have compared our predicted subcellular localization data for Arabidopsis proteins (the best annotated in our sequence set from photosynthetic organisms) to the data residing in the SUBA3 database (the SUBcellular localization database for Arabidopsis proteins) [72]. In all instances our predictions are supported. The proteins At2g30170.1, At4g33500.1, At5g66720.1 have all been shown to be chloroplast-localized, by MS/MS analysis. In addition, At2g30170.2 is predicted to be cytoplasmic, and At4g16580.1 is predicted to be mitochondrial, by SUBAcon [73], a Bayesian classifier working on all available evidence (At2g30170.2 [SUBAcon: cytosol 0.937]; At4g16580.1 [SUBAcon: mitochondrion 0.955]).

### Examination of plant PP2C7 gene expression and gene co-expression network data

To attempt to gain insights into the possible function(s) of plant PP2C7 genes we accessed, assembled and distilled a variety of publically available gene expression data. These are summarized in Table 1 and Table G in S1 File, which present information about tissue gene expression and hormone responses, and Table H in S1 File, which summarizes information gleaned from descriptions of plant gene co-expression networks. Arabidopsis At2g30170 and its various gene homologues in several dicot and monocot species are expressed almost exclusively in obviously photosynthetic locations–the only possible exception being one *Populus trichocarpa* homologue which is reported to be expressed maximally in seedlings in the dark. The expression pattern begins to be more complicated for the group of homologues related to Arabidopsis At4g33500. That gene, as well as homologues from both dicot and monocot species, continue to show maximal expression in photosynthetic tissues. However probes for rice homologues show maximal or near maximal expression in root or floral structures. In addition, probes for both rice and maize genes show stress responses—abiotic stress for loci Os03g09220 (Os03g0192500) and ZmPP82 (GRMZM2G071087). This increased complexity of gene expression response continues for the homologues of Arabidopsis genes At4g16580 and At5g66720. It is most unfortunate that probes for these two genes are not on the standard Affimetrix gene chip. In other species some gene loci continue to manifest maximal expression in photosynthetic tissues. However other distinct gene loci in both dicots and monocots show maximal expression in non-photosynthetic or specialized tissues, including root and various reproductive structures. For the “Dicot” PP2C7 gene group, limited data (PlaNet [60]) shows expression of individual homologues being limited exclusively to non-photosynthetic or specialized tissues: root, female catkins, male catkins. Finally, some data suggest that genes in the “Monocot” PP2C7 gene group may be only very weakly or even non-expressed (maize [62]; rice [23]). However, other rice data (PlaNet [60]) is consistent with the Populus pattern, with maximal expression in seed and coleoptile.

**Table 1:** Summary of plant PP2C7 gene expression data.

PP2C7 Gene Group	Species	Probe/Gene	Max Photosynthetic Tissue Expression	Max Non-Photosynthetic/Specialized Tissue Expression
**30170**	Arabidopsis	At2g30170	21 day shoots	
	Medicago	Mtr.27410.1.S1_at/Medtr3g031360.1	Leaf 28 days	
	Tomato	LesAffx.35972.1.S1_at/Solyc06g007350.2.1	Cotelydon	
	Populus	PtpAffx.13701.1.A1_at/POPTR_0001s2893	Mature leaf	
		Ptp.5027.1.S1_at/POPTR_0001s2893		Seedlings in dark
	Rice	Os.5408.1.S1_at/LOC_Os01g07090.1	Shoot	
	Barley	Contig13371_at/MLOC_19725.2	Seedling leaf	
	Maize	ZmPP46 (GRMZM2G003096)	V5_Tip of stage 2 leaf	
**33500**	Arabidopsis	At4g33500	Shoot 21 days	
	Medicago	Mtr.7881.1.S1_at/medtr2g008670.1	Leaf 28 days	
	Tomato	Les.759.1.A1_at/Solyc01g105020.2.1	Seedling	
	Rice	Os.11601.1.S1_at/LOC_Os10g22460.1		Root
		Os.46691.1.A1_x_at/LOC_Os10g22460.1		Root 7d seedling
		17825/LOC_Os10g22460/Os10g0370000		Root, vegetative
		7676/LOC_Os03g09220/Os03g0192500	Leaf Blade	Lemma, 7mm floret
		43513/LOC_Os03g09220/Os03g0192500	Leaf Blade	Lemma, 7mm floret
		Os.7974.2.S1_at/LOC_Os03g09220.2		inflorescence (P6 22-30cm)
	Maize	ZmPP82 (GRMZM2G071087)	V5_Tip of stage 2 leaf	
		ZmPP82 (GRMZM2G071087)	VT_Thirteenth leaf	
**16580**	Arabidopsis	At4g16580/gene not on chip		
		At5g66720/gene not on chip		
	Populus	PtpAffx.152041.1.S1_at/POPTR_0014s0423		Root
	Medicago	Mtr.48583.1.S1_at/Medtr5g019790.1	Leaf 28 day	
		Mtr.39542.1.S1_at/Medtr5g019790		Pod 21 day
	Tomato	LesAffx.68099.1.S1_at/Solyc12g089091.1.1		pericarp walls (flesh)
		LesAffx.5120.1.S1_at/Solyc07g064310.1.1		Skin (exocarp)
	Soybean	Gma.12590.1.A1_at/Glyma16g25560		Globular stage embryo
		GmaAffx.83171.1.S1_at/Glyma11g07030		4d seedling root
	Rice	Os.9591.1.S1_at/LOC_Os03g59470.1	Mature leaf	
		40036/LOC_Os03g59470(Os03g0809300)	Leaf blade, vegetative	inflorescence (lemma, 7mm floret)
	Maize	ZmPP78 (AC210013.4_FG011)	V5_Tip of stage 2 leaf	
		ZmPP23 (GRMZM2G134227)		16DAP endosperm
	Wheat	Ta.592.1.S1_at/gb|BQ172270		Mature anthers
**Dicot**	Populus	PtpAffx.200357.1.S1_at/POPTR_0001s1084		Female catkins
		PtpAffx.201351.1.S1_s_at/POPTR_001s3900		Root
		PtpAffx.222293.1.S1_x_at/POPTR_005s17490		Male catkins
		also POPTR_0017s05320		
**Monocot**	Rice	OsAffx.24684.1.S1_at/LOC_Os02g42250.1		seed (S5 21–29 dap)
		OsAffx.12452.1.S1_at/LOC_Os02g42270.1		coleoptile, 4d aerobic

Plant gene expression data was accessed from the sources detailed in “Materials and Methods”. Most data is from PlaNet ([60]; http://aranet.mpimp-golm.mpg.de/). Tomato data is from Genevisible/Genevestigator ([61]; http://genevisible.com/search). Maize data is from [62]. Additional data on hormone responses are given in Table G in S1 File.

Within the co-expression network of Arabidopsis At2g30170 are a number of genes encoding proteins from chloroplast photosystems, including the protein kinase STN8 (STATE TRANSITION 8) (At5g01920), which is involved in regulation of light adaptation [74]. In addition, there are several encoding proteins characteristic of stress responses. This pattern is to a large extent shared by the co-expression network of the rice homologue in the 30170 group (Os01g07090 [Os01g0164600]). However, in addition there are several genes pertaining to Ribulose bisphosphate carboxylase (RuBisCO), suggesting a possible role in regulation of carbon fixation.

For Arabidopsis At4g33500 there is a first-order linkage in the reported gene co-expression network with the locus At4g18240, which encodes SSIV (STARCH SYNTHASE 4). Deeper within the co-expression network appear genes for several other starch metabolic enzymes as well as the starch metabolism regulating protein phosphatases SEX4 (STARCH-EXCESS 4) (At3g52180), LSF1 (LIKE SEX4 1) (At3g01510) and LSF2 (LIKE SEX 4 2) (At3g10940) [75]. These co-expression patterns suggest that a major function of the At4g33500 gene and protein may be regulation of starch metabolism. This inference is supported by additional evidence. First, the expression pattern of At4g33500 is clearly diurnal, being visibly similar to that of other well-characterized genes involved in starch metabolism. This data is summarized in Fig B in S2 File. Furthermore, promoter element analysis of At4g33500 demonstrates the presence of two instances of the EVENINGAT element, well known in diurnal genes [76] and also present in the promoters of starch metabolic genes At1g70820 (PGM2 [phosphoglucomutase 2]), At5g64860 (DPE1 [DISPROPORTIONATING ENZYME 1]), and At3g29320 (PHS1 [plastidial phosphorylase isozyme 1]) (data not shown). An additional promoter element in At4g33500 was first reported in starch branching enzyme 1 in maize endosperm [77]. A functional interaction between the Arabidopsis PP2C7 At4g33500 protein and SSIV is made more probable by their physical characteristics. We have previously reported SSIV to have extensive coiled-coil regions [78]. Our unpublished data show that At4g33500 also has a region of strong coiled-coil potential. Such regions have often been demonstrated to mediate protein-protein interactions in other systems [79], [80]. The gene co-expression network of At4g33500 contains, in addition, a number of genes encoding chloroplast photosystem components, and genes for proteins likely involved in stress responses. These may suggest additional, perhaps secondary functions for this gene.

One rice homologue in the 33500 gene group (Os10g22460 (Os10g0370000) has a first-order linkage in its gene co-expression network with a starch synthase (SSII-B (Os02g51070)), suggesting the possibility of a conserved function in regulation of starch metabolism. This is consistent with a markedly diurnal pattern of expression for this gene (data not shown). However, this is the only starch metabolic enzyme in this network, suggesting that for this gene other functions may be more important. There are several putative stress-response genes, including the rice homologue of the well-characterized Arabidopsis protein phosphatase ABI2 (ABA INSENSITIVE 2). This suggests the possibility of a role for this rice PP2C7 gene in the regulation of abscisic acid responses. Most numerous are genes and proteins which would be expected to be involved in signal transduction processes, including several protein kinases and a wide assortment of putative transcription factors. This might indicate a major shift toward regulation of gene transcription, perhaps associated with the prominence of root expression for this gene.

Another rice homologue in the 33500 gene group (Os03g09220 (Os03g0192500)) has a first-order linkage in its gene co-expression network with Os05g47560 (Os05g0549100), the rice homologue of the Arabidopsis regulatory kinase STN7 (STATE TRANSITION 7). In Arabidopsis this protein is involved with STN8 in the light acclimation response, where the latter is opposed by the PP2C7 At2g30170 [74]. This suggests that in rice the PP2C7 gene homologues of At2g30170 and At4g33500 may share in light acclimation regulation. In addition the co-expression network of Os03g09220 has many genes encoding proteins likely to be involved in biotic and abiotic stress responses, suggesting a possible regulatory role. Finally, there are once again a large number of genes expected to be involved in signal transduction, including protein kinases, protein phosphatases, and various putative transcription factors. Their presence in the gene co-expression network may be related to the shift toward high levels of Os03g09220 gene expression in a specialized reproductive structure.

The gene co-expression network for the rice homologue in the 16580 group (Os03g59470 (Os03g0809300) is novel in that it contains several genes involved in various aspects of vesicle transport. This network is also dominated to an even greater degree than seen previously by a wide assortment of signal transduction proteins. It is interesting that this gene shows substantial expression levels across several reproductive structures (inflorescence, anther, pistil, lemma, palea, ovary) (data available at RiceXPro [63]; http://ricexpro.dna.affrc.go.jp/)), a characteristic not seen previously, and consistent with maximal expression patterns of PP2C7 genes from the 16580 group in various other species. Taken together these data suggest that the rice 16580 group homologue gene may be involved in regulation of the various complex aspects of reproductive structure development.

### Relatively few PP2C7s have previously-characterized accessory protein domains, but most plant sequences have distinctive novel N-terminal motifs

Functional inferences about novel proteins are often facilitated by knowledge of their domain architectures. Possession of a well-characterized protein domain makes it very likely that a given function is exercised by that protein [21]. We therefore characterized the domain organization of the PP2C7 proteins in our dataset by a variety of standard procedures. Despite variations in length, the domain organization of most PP2C7 sequences appears simple, with no known accessory domains being detected in ~91% of the cases (228/251). The typical architecture has the PP2C7 domain at the carboxy terminus of the sequence. The presence of “empty” N-terminal sequence flanking the phosphatase domain of many PP2C7 proteins could sometimes indicate annotation errors and incorrect gene models; however, there is also the potential for the existence of novel, uncharacterized accessory domains, particularly in plant sequences.

To test this hypothesis, we subjected the amino terminal regions of plant PP2C7 sequences (lacking previously-characterized accessory domains) to motif analysis. The results are shown as sequence logos in Fig C in S2 File. (these are presented as conventional sequence alignments in Fig D in S2 File.). When the entire sequence set is tested, there is detectable but weak similarity present in a motif of 40 characters. This weak overall similarity is to be expected given the deep evolutionary divergence between the two assemblages containing plant sequences presented above. Furthermore, most of the previously described phylogenetic tree groups (At2g30170, At4g33500, At4g16580, Monocot) possess a distinctive sequence motif. The exception is the Dicot group. Despite having an average length comparable to that of the At2g30170 group and Monocot group (72.7 residues, versus 75.6 and 80.8, respectively), the Dicot group upon motif inference produced a result only 7 amino acids long (data not shown). When the motifs presented for the various groups were used to search the NCBI non-redundant protein database, a sampling of hits with scores greater than 50% of the maximum revealed that they were all from plants. This suggests that these motifs are plant-specific, and is consistent with them playing a regulatory role in PP2C7 protein function.

Amongst the relatively few previously-characterized accessory domains detected, several were putative protein interaction domains (LIM, PLAT, CBS_pair, WW, coiled-coil) (see Table I in S1 File for a compilation of all accessory protein domain data and domain annotation notes). One example of a dual calmodulin-binding domain protein was found. Two algal sequences (one from *Chlamydomonas*, one from *Volvox*) were found to possess a TIR (Toll/Interleukin 1 Receptor) domain. This domain is involved in innate immunity in animal species, and similar domains in plants are thought to be involved in defense responses [81]. Two algal proteins (one from *Chlamydomonas* and one from *Volvox*) were found to possess a CBM20 (carbohydrate binding motif) domain. This is consistent with a possible involvement of these sequences in some aspect of carbohydrate metabolism, potentially in regulation [75].

Perhaps the most interesting accessory domains detected were those likely to be involved in nucleic acid binding. A SAP domain was detected in three green algal sequences (from *Bathycoccus*, *Ostreococcus lucimarinus* and *Ostreococcus tauri*) and in the Rhizarian *Bigelowiella*. This is a putative DNA/RNA binding domain [82]. PIN_Utp23 domains were found in sequences from four fungal species (*Grosmannia*, *Metarhizium*, *Verticillium alfalfae* and *Verticillium dahliae*). These domains in fungal proteins are involved in nuclear small rRNA processing [83].

### Search for possible PP2C evolutionary gene paths between Bacteria and Eukaryotes

The topology of our previously presented phylogenetic trees (Figs 1, 2 and 3)–bacterial Group IIs most closely related to eukaryotic PP2C7s and bacterial Group Is most closely related to eukaryotic PP2Cs–suggests the likelihood of genetic exchange between the Bacteria and Eukaryotes. We next sought further evidence which might clarify the nature of these possible events.

We first set about attempting to determine whether it is possible to define more precisely which subgroup of bacterial Group IIs might be most closely related to the PP2C7s, and therefore provide a link to putative ancestral sequences. From our set of confirmed bacterial Group II sequences we selected a large set (297 sequences) representing a very broad range of bacterial phyla (16 phyla). We began utilizing both phylogenetic trees and HMM-HMM comparisons (HHAlign) to examine similarities between bacterial Group II subgroups and a set of eukaryotic PP2C7 sequences. It quickly became apparent that bacterial Group II sequences are heterogeneous with respect to their composition at classic PP2C Motif 5. The most common motif type contains the key residues "GH" (~60 percent of our large bacterial Group II set). However, we encountered a subgroup of sequences (~5% of the total) which possess "GN" at this motif position. Phylogenetic tree analysis followed by HMM-HMM comparisons showed that the bacterial Group II subgroup containing the Motif 5 "GN" signature was more highly similar to the eukaryotic PP2C7s than were the remainder of the "bulk" bacterial Group II set (see Table J in S1 File for summary HHAlign data).

We expanded this analysis to include an even larger bacterial “GN” Group II sequence set in two stages. First, we utilized the bacterial Group II sequences automatically collected under the Pfam model PF07228 (SpoIIE). At the time of our access, this set contained 6326 sequences. (It should be noted that this automated set probably contains a substantial fraction of contaminating bacterial Group I sequences, as determined by our HHPred analysis of a small random sequence sample). We made an HMM based on our set of bacterial “GN” Group II sequences (95), and searched this Pfam sequence set. Consistent with our earlier results, we recovered a sequence subset (276 [4.36% of the total]) containing the "GN" Motif 5 signature. We next used our “GN” Group II HMM to search the UniProt database of all bacterial sequences (~3.23E7 sequences). This process resulted in a final consolidated set of 414 "GN" Group II sequences. The UniProt identification numbers and taxonomic distribution of these sequences is summarized in Table K in S1 File.

Preliminary multiple sequence alignments and phylogenetic trees analyzing the bacterial GN PP2C set showed sequence segregation into two large assemblages. One is formed by sequences which cluster in the neighbourhood of the well-studied environmental stress phosphatase RsbX of the model organism *Bacillus subtilis* (BsP17906). Many of these sequences are annotated in UniProt as RsbX homologues. We have designated these “More RsbX-Like”. A second more distant sequence assemblage consists of sequences which we designate “Less RsbX-Like”. Some of these sequences (but a markedly lower portion than previously) are also annotated as RsbX homologues. We next compared sequences in these two assemblages to PP2C7s by HHAlign. The results are summarized in Table L in S1 File. It is clear that the “More RsbX-Like” sequences have a much stronger similarity to the PP2C7s.

We next produced a multiple sequence alignment encompassing the “More RsbX-Like” bacterial GN Group II sequences and the entire PP2C7 sequence set (shown in Fig A in S2 File, Panel 5). Phylogenetic tree inference produced the data presented as Fig E in S2 File. A cluster of GN Group II sequences which is most closely related to the PP2C7 set is derived from the order Myxococcales (fruiting gliding bacteria) of the δ-Proteobacteria. Immediately adjacent is another cluster of sequences from the Proteobacteria, comprising representatives from the α,β, and γ classes. We next removed the bacterial sequence cluster from Myxococcales (alignment shown in Fig A in S2 File, Panel 2), and inferred a new phylogenetic tree. The result is presented as Fig 4. Now the α-proteobacterial sequences from the previous α,β,γ-proteobacterial cluster are most closely related to the eukaryotic PP2C7s. These results indicate that one or the other of these two bacterial sequence clusters (δ-proteobacterial or α-proteobacterial) is likely descended from the ancestral group for the eukaryotic PP2C7s.

To attempt to further differentiate between these two possibilities, we next investigated the relationship between the “More RsbX-Like” bacterial GN Group II set and a comparison group composed of eukaryotic PP2Cs (lacking PP2C7s) from human and *Arabidopsis*. Our reasoning in formulating this comparison set was as follows: since our previous phylogenetic trees (Figs 1, 2 and 3) had shown that eukaryotic PP2Cs were derived from a common ancestor with the PP2C7 lineage (e.g. Point 2, Figs 1 and 2), then a direct comparison between the PP2C sequences and the “More RsbX-Like” bacterial GN Group II sequences should give a most closely related bacterial subgroup consistent with the likely origin of the PP2C7s. The results of this analysis are presented as Fig 5 (alignment shown in Fig A in S2 File, Panel 3). Consistent with this prediction, the same sequence cluster from α,β,γ-Proteobacteria was again most closely related to the eukaryotic PP2Cs, with the Myxococcales set being in a more distant tree position. We infer therefore that eukaryotic PP2C7s likely originated from an ancestral sequence lineage currently represented in the α,β,γ-proteobacterial cluster, most likely in the α-proteobacterial component.

In order to investigate further the relationship between eukaryotic PP2Cs and bacterial Group I sequences, we first built a much larger sequence alignment containing substantial numbers of bacterial Group I, eukaryotic PP2C, and bacterial Group II sequences, in combination with a large PP2C7 set. This large alignment contained the eukaryotic PP2C sets from human and *Arabidopsis*, plus a substantial number of bacterial Group I and bacterial Group II sequences, chosen at random from a large number of phyla. We noted in the resulting phylogenetic tree that there were sequences from bacterial genera (*Protochlamydia*, *Parachlamydia*) which were deeply embedded within the eukaryotic PP2C clade. HMM-HMM analysis by HHPred showed that these sequences were much more closely related to the solved structures of eukaryotic PP2Cs than to those of solved bacterial Group I sequences. We therefore utilized Hidden Markov Models made from large alignments of eukaryotic PP2C sequences to search a database of all UniProt bacterial sequences. High scoring hit sequences were then subjected to HHPred analysis. This procedure obtained further sequences from the bacterial genera *Waddlia* (Chlamydia), *Legionella* (γ-Proteobacteria) and an “uncultured bacterium” with strong similarity to eukaryotic PP2Cs. The HHPred analysis which confirms that these nine sequences, though present in bacterial species, are much more closely related to the structures of solved eukaryotic PP2Cs than they are to those of solved bacterial Group I structures, is presented in Table M in S1 File. A final large tree encompassing expanded sequence sets from eukaryotic PP2Cs (human and *Arabidopsis*), bacterial Group Is, and bacterial Group IIs, along with a substantial eukaryotic PP2C7 component, is presented in Fig 6 (alignment shown in Fig A in S2 File, Panel 4). It can be readily seen that the typical topology observed previously is seen once again here—that is, bacterial Group II sequences (Node B) are most closely related to the PP2C7 sequences (Node A) (groups connected by Node C), and the bacterial Group I sequences (Node D) are most closely related to the eukaryotic PP2Cs (Node E) (groups connected by Node F). This thus confirms the findings of smaller sets of comparison sequences, and rules out any possibility of phylogenetic tree artifacts arising from biased taxon sampling effects. Furthermore, it can be seen that deeply embedded within the eukaryotic PP2C clade is the set of nine sequences from bacterial genera discussed above. In the Discussion we will return to the possible evolutionary significance of these “eukaryotic-like” PP2C sequences in current bacterial genera.

### Conservation of sequence motifs throughout PP2C evolution

Early work analyzing the sequences of PP2C proteins showed that this group is characterized by eleven conserved motifs, containing a set of conserved amino acids including four aspartate residues (in Motifs 1, 2, 8, and 11) [84]. Since then, a number of PP2C structures have been solved, and they share the common architecture of an alpha-beta sandwich, consisting of two interior parallel beta sheets, surrounded by exterior alpha helices. Comparison of the secondary structure of several solved PP2Cs yields the diagram shown in Fig F in S2 File. Fig G in S2 File shows a large reference multiple sequence alignment of PP2C7 sequences. This alignment shows that these multiple aspartic acid residues are nearly absolutely conserved. Fig 7 shows a multiple sequence alignment guided by superposition of several solved PP2C structures. The conservation of aspartate residues in Motifs 1, 2, 8 and 11 (highlighted in red) is evident. In addition, there is another aspartate, present in Motif 5 (highlighted in purple), which is conserved in some but not all sequences. This residue is present in the PP2C7s, the bacterial Group I and the eukaryotic PP2C sequences, but it is missing in the bacterial Group II sequences. This Motif 5 aspartate is also present in all but two of the PP2C7s in the large reference alignment of Fig G in S2 File. The inset phylogenetic tree in Fig 7, with the same topology seen earlier in Figs 1, 2, 3, and 6, illustrates an evolutionary model for PP2C conserved aspartate residues. Two sets of aspartates (at Motifs 1 and 2; 8 and 11) appear to be an ancestral trait for PP2C proteins. The later acquisition of an additional aspartate at Motif 5 appears to be a more derived character, appearing first in the common eukaryotic PP2C ancestor (Point 2 in Figs 1 and 2), and continuing in the PP2C7 lineage, in eukaryotic PP2Cs and in bacterial Group I sequences. This model is supported by our discovery of the subset of bacterial Group II PP2C sequences which contain the GN signature at Motif 5. This group is represented in the alignment of Fig 7 by Bs3W40 (RsbX from *Bacillus subtilis*) and Mt3ZT9 (the RsbX homologue from *Moorella thermoacetica*). We have presented above data strongly suggesting that the GN Group II subgroup, especially those from ancient α,β, and γ-Proteobacteria, are ancestral to eukaryotic PP2C7s. Our stepwise model of PP2C sequence change at Motif 5 postulates the key acquisition of the subsequently conserved aspartate (D) residue here. Since asparagine (N) and aspartate (D) are traditionally considered as conservative amino acid substitutions, this would provide a shorter mutational path to the modification of Motif 5 in the common eukaryotic PP2C ancestor than would the more numerous “bulk” bacterial Group II signature with a conserved histidine (H) at this position.

## Discussion

### The Evolutionary Trajectory of PP2C7 Genes and Proteins in Plants

Our phylogenetic results clearly demonstrate a deep and ancient division in genes encoding PP2C7 proteins, with the presence of two distinct multi-species assemblages of genes: “Assemblage A” and “Assemblage B” (Fig 3). Strictly speaking, the topology of our tree does not allow us to distinguish which of these groups is the more ancient. However, the fact that the great predominance of algal sequences available (17 of 24) is in Assemblage A, suggests that this is the more basal group. This is consistent with both the subcellular localization data we present, which is predominantly chloroplastic, and with the gene expression data we report, where maximal expression seems to be limited to photosynthetic tissue, suggesting a conserved ancient photosynthesis-related function. This is consistent with the function of the Arabidopsis gene At2g30170 reported from results of a mutation screen. The protein product has been named PHOTOSYSTEM II CORE PHOSPHATASE (PBCP), and is localized to thylakoid membranes within chloroplasts. This protein is required for efficient dephosphorylation of PSII core proteins, and is involved in the folding of thylakoid grana. It acts as the antagonist to the protein kinase STN8, with which it appears to be important for a normal light acclimation response [74]. The characteristics of the co-expression data we report here for the rice 30170 gene group homologue (many photosystem proteins, the rice homologue of STN8) make it likely that in rice this role in light acclimation is conserved. However, characteristics of the co-expression networks for both Arabidopsis At2g30170 and its rice homologue suggest that additional, as yet uncharacterized functions may exist for these genes.

The fact that Assemblage B contains relatively few algal sequences suggests that it is the more recently diverged group. Furthermore, there is a clear phylogenetic cluster pattern within this assemblage showing a progressive diversification: first the At4g33500 group, then the At4g16580 group, then finally the “Dicot” and “Monocot” gene groups. There is also an increasing divergence of predicted subcellular localization, with the At4g33500 group predicted to be about half chloroplastic and half not; the At4g16580 group predicted to be predominantly mitochondrial; and the “Dicot” and “Monocot” groups predicted to be cytoplasmic. These patterns also correlate with the increasing divergence of gene expression patterns which can be discerned from the maximal gene expression and gene co-expression network results we report. The protein product of the Arabidopsis At4g33500 locus has been demonstrated to be chloroplastic by experiment [70], though at present its function remains unknown. The coiled-coil character of this protein plus the gene expression and co-expression patterns we report here make it possible that a major function of At4g33500 is in regulation of starch metabolism. The pattern of relationships in the co-expression network for this gene suggests additional possible functions as well. Co-expression patterns indicate that while regulation of some aspects of starch metabolism may also be a function of the rice 33500 homologue Os10g22460, other functions may have become predominant for this gene, which is consistent with its shift of maximal expression to roots. A shift in predicted subcellular protein localization plus the prominence of stress responses are characteristic of the other rice 33500 homologue Os03g09220. In a published report [85] one cloned isoform (which is either isoform 2 or isoform 3, and therefore predicted to be cytoplasmic) was named *OsBIPP2C1* (*Oryza sativa* BTH-Induced PP2C1), as the expression of this gene is induced by benzodiathiazole (BTH), a signalling molecule in plant disease resistance responses. In addition, gene expression is induced by several types of abiotic stress. Overexpression of this gene in transgenic tobacco conferred enhanced resistance to viral and oomycete infection, as well as enhanced abiotic stress resistance.

This trend toward greater complexity of expression continues in the members of the At4g16580 group, where in addition to photosynthetic tissue expression, maximal expression is reported for several members in non-photosynthetic or specialized tissues. The rice 16580 homologue Os03g59470 is expressed maximally in the leaf blade, but at nearly equal levels in lemma, and at substantial levels in several other reproductive structures. Taken together, the data indicate that PP2C7 genes in the 16580 group may be important regulators of various aspects of reproductive organ development.

Finally, though the experimental data is limited, it appears that for the “Dicot” and “Monocot” gene groups, the gene expression patterns (exclusively in non-photosynthetic and specialized tissues), cellular localization predictions (cytoplasmic) and phylogenetic position (most highly derived clusters) consistently indicate a high degree of divergence from ancestral plant PP2C7 functions. The possibility needs to be taken seriously that the “Monocot” genes (at least in maize and rice) may be pseudogenes. It is both a challenge and opportunity for future research to attempt to fill in this outline with more detailed characterization of individual plant genes and their proteins in a variety of species.

### PP2C Origins and Diversification

The PP2C enzymes play defined roles in various aspects of both bacterial and eukaryotic signal transduction and metabolic pathways [25, 33, 86, 87]. The origin and evolutionary diversification of PP2Cs has been controversial. One study, which first characterized bacterial Group I and II sequences, proposed that both groups arose in Eukaryotes and were spread to Bacteria by lateral gene transfer (LGT) [71]. Other studies, one which initially defined the classic PP2C sequence motifs [84] and another which did an extensive phylogenomic analysis of bacterial Group II sequences [88], concluded that they had a deep and ancient origin in Bacteria. We will argue that each of these earlier views contained an element of truth. Characterization of the new PP2C7 clade adds an additional crucial element to the phylogenetic tree of all PP2C groups, allowing a more complete resolution of the pattern of PP2C evolution.

In a previous study of PP2C sequence evolution in metazoans [34], the “TA-PP2C” group (T-cell activated) was utilized as an outgroup in an unrooted phylogenetic tree (their Fig 1). These sequences in fact correspond to the animal subset of the much more extensive PP2C7 sequence clade we characterize here. Our rigorous analysis utilizing rooted phylogenetic trees (Figs 1 and 2) confirms the distinctive nature of PP2C7 sequences from a broad range of Eukaryotes, including the large expansion in photosynthetic species. In addition, our tree topologies demonstrate a relationship between bacterial Group II sequences and PP2C7s, with the root clearly placed within Bacteria. Later diversification of a common eukaryotic ancestor sequence type eventually led to both PP2C7s and the conventional eukaryotic PP2Cs.

Mitochondrial endosymbiosis at the origin of Eukaryotes is a well-established mechanism for the importation of a variety of bacterial genes. We have previously demonstrated that this likely occurred for protein phosphatases of the “bacterial-like PPP” type [36]. Our phylogenetic tree data from both PP2C7s (Fig 4) and eukaryotic PP2Cs (Fig 5) support a cluster of bacterial Group II sequences (possessing GN at classic motif 5) from α,β, and γ-Proteobacteria (especially the α-proteobacterial component) as being descended from ancestors most likely to have given rise to modern PP2C7s. The simplest and most biologically robust hypothesis is to assume that it is in fact the α-proteobacterial gene lineage which entered Eukaryotes, at the ancestral mitochondrial endosymbiosis, and which then diversified to form both the modern PP2C7s and the other eukaryotic PP2Cs. This is consistent with a growing body of research showing that various eukaryotic signaling proteins [89, 90], as well as proteins of innate immunity and apoptosis [91], have their origin in Bacteria.

It is intriguing that in the phylogenetic tree in Fig E in S2 File it is sequences from the Myxococcales (gliding fruiting bacteria) which are the most closely related to the PP2C7s. We interpret this as these bacterial sequences being the sister group of the eukaryotic sequences, arising from a common α-proteobacterial ancestry. We recently demonstrated a similar relationship in two classes of “bacterial-like PPPs” [36].

Our phylogenetic tree data (Fig E in S2 File, Fig 4) and HMM-HMM comparison data (Table L in S1 File) both show that the PP2C7s are most closely related to bacterial GN Group II sequences of the RsbX type. In fact, several sequences in the Myxococcales cluster and the α,β,γ-proteobacterial cluster related to the PP2C7s are annotated as RsbX’s. RsbX proteins are conserved phosphatases involved in the widely distributed bacterial “stressosome”. This is a modular multi-component assembly which through kinase and phosphatase mediated changes in protein phosphorylation effects downstream signaling, either directly through alternative sigma factor changes in gene expression, or indirectly via signalling enzymes such as diguanylate cyclases [92, 93].

The phylogenetic framework for PP2C evolution which we have established clearly shows the close relationship between bacterial Group I sequences and eukaryotic PP2Cs (Point 3, Figs 1 and 2), and the origin of the latter within Eukaryotes from a common ancestor with PP2C7s (Point 2, Figs 1 and 2). This strongly suggests that a gene transfer must have occurred from Eukaryotes to Bacteria, in order to produce through subsequent diversification the modern bacterial Group I sequences. Our data provide evidence to support such a possibility.

We observed chlamydial and *Legionella* sequences within the Eukaryotic PP2C clade, which possess far higher similarity to eukaryotic than to bacterial sequences. It has been previously observed and reported on a number of occasions that photosynthetic Eukaryotes contain a number of sequences most closely related to those from Chlamydiae. These findings were initially interpreted to suggest lateral gene transfer (LGT) from plants or plant-related organisms to chlamydial ancestors [94–96]. However, a growing body of evidence now suggests that instead, chlamydial organisms were co-inhabitants with cyanobacteria of the ancient heterotrophic Eukaryote which subsequently captured the cyanobacterium to form the primary chloroplast endosymbiont [97–99]. The presence of closest similarity between modern chlamydial and eukaryotic photosynthetic organism sequences in several enzymes critical to photosynthate transport and starch metabolism suggests that the chlamydial gene contributions were critical to the metabolic integration between the cyanobacterium and the eukaryotic host [100, 101]. An example is the similarity between *Protochlamydia* and *Parachlamydia* sequences for starch debranching enzymes and those of photosynthetic Eukaryotes [100]. It has been shown that organisms of the genera *Protochlamydia* and *Parachlamydia*, as well as certain *Legionella* species (such as *L*. *drancourtii*) are currently inhabitants of eukaryotic amoebae, where they form a stable community resistant to host digestion. These organisms, in contrast to the tendency for genome reduction in modern obligate intracellular parasites, have retained enlarged genomes, having accumulated many genes, including eukaryotic genes, which were apparently laterally transferred to their ancestors in the past from other organisms [94, 102]. Hence these “amoebae-resistant microorganisms” are capable of acting as a genetic repository, preserving a record of past lateral transfer events. The simplest interpretation of the “eukaryotic-like” sequences that we have observed in these present bacterial genera is that they are products of past lateral transfer events from Eukaryotes. Were similar transfers to have occurred in the distant past from ancient Eukaryotes to ancestral chlamydiae and *Legionella*, their subsequent dissemination amongst other bacteria, followed by diversification, would account for the production of the modern bacterial Group I set of PP2Cs.

### Multi-domain PP2C7 proteins may be evolutionarily or functionally significant

Most of the relatively rare previously-characterized accessory domains we detected in PP2C7 protein sequences have an isolated and scattered phylogenetic distribution, suggesting relatively recent evolutionary adaptations by individual organisms. However, a few cases we detected deserve closer consideration. The CBM20 domains detected in two algal sequences (*Chlamydomonas* and *Volvox*) may suggest a role, perhaps regulatory, in starch metabolism. The phylogenetic distribution of the SAP putative DNA-binding domain containing sequences suggests recruitment of the PP2C7 domain into a nucleic acid binding protein fairly early in evolution. The three algal species containing them all belong to the early-diverging class Mamiellophyceae. Furthermore, the presence of such a sequence in the Rhizarian *Bigelowiella* is consistent with the secondary green algal endosymbiotic origin of this organism [103, 104]. Finally, the presence of PP2C7 domains in sequences together with putative rRNA processing PIN_Utp23 domains in several fungal species may be significant. Three organisms involved are destructive plant pathogens (*Verticillium alfalfae*, *Verticillium dahliae* [crop pathogens] [105]); *Grosmannia clavigera* [lodgepole pine pathogen] [106]. It is conceivable that the recruitment of this domain combination might be significant to the pathogenic lifestyle. If so, this protein might represent a novel drug target.

### Evolution of PP2C metal binding and catalysis

There has been recent controversy surrounding the architecture of PP2Cs required for catalysis. The classic solved structure of a eukaryotic PP2C (human PP2Cα [PDB: 1A6Q]) featured two bound metal ions [3]. This involved primarily classic PP2C motifs [84] 1, 2, 8 and 11. More recently, solved structures from bacterial Group I PP2Cs (MtPstP [PDB: 1TXO], SaSTP [PDB: 2PK0], MspP [PDB: 2JFR], tPphA [2J82]) have revealed three bound metal ions [12, 15, 16, 107]. Binding of the third metal ion requires an aspartate in classic Motif 5. Elegant biochemical experiments clearly demonstrate that for the bacterial Group I PP2Cs, but also surprisingly for the prototype human PP2Cα, this Motif 5 aspartate is essential for catalytic activity [17]. This implies that under physiological conditions eukaryotic PP2Cs also have three bound metal ions. This has been confirmed in one instance by the recently solved structures of an *Arabidopsis* PP2C, HAB1 (PDB:3QN1; 3KB3), which contain three bound metal ions [13, 14]. More recently, detailed thermodynamic measurements confirmed the presence of a third, weakly bound metal ion in human PP2Cα [18]. This report also showed by mutagenesis and kinetic analysis that an additional aspartate in Motif 8 (four positions downstream of the classic conserved aspartate residue) is required for efficient third metal binding and catalysis.

The stepwise evolutionary model we presented as the inset in Fig 7, previously examined in terms of aspartate sequence conservation, thus can now also serve for the evolution of metal binding pockets capable of sponsoring phosphoprotein catalysis. Our phylogenetic analysis indicates that the presence of an aspartate at Motif 5 would be an innovation of the common eukaryotic ancestor PP2C sequence (Point 2, Figs 1 and 2) and we postulate this would have conferred the ability to bind three metal ions. This property could then be inherited by all subsequent progeny lineages. Three metal binding pockets would thus be predicted for PP2C7s, eukaryotic PP2Cs, and bacterial Group I PP2Cs. This hypothesis readily explains the recent puzzling apparent similarity of action of eukaryotic PP2C and bacterial Group I PP2C catalysis. In addition, the alignment in Fig 7 shows that the supplemental conserved aspartate in Motif 8 identified by Tanoue [18] as contributing to third metal binding in human PP2Cα is conserved in PP2C7s (residues highlighted in orange). An examination of our very large sequence alignments shows that this aspartate is only sporadically present in bacterial Group II PP2Cs (frequency ~16% [~270 of 1696] in the total original HMM search sequences; frequency ~6% [~25 of 417] in “GN” sequences), whereas it is nearly universally conserved (altered in only two full length sequences) in the large reference alignment of Fig G in S2 File. This strongly suggests that this residue first appeared consistently in the common eukaryotic PP2C ancestor sequence, and was subsequently inherited by progeny lineages. This reinforces the likelihood that PP2C7 proteins will also prove capable of binding a third metal ion.

This stepwise model predicts that bacterial Group II PP2Cs, since they lack the key aspartate in Motif 5 (and also in general the supplemental aspartate residue in Motif 8), would be unable to form a third metal binding pocket, and would thus only be capable of binding a maximum of two metal ions. A small number of such proteins have been studied, and confirmed to act as protein phosphatases [84, 93, 108]. At present there are several relevant solved structures of bacterial Group II PP2Cs (PDB: 3F79, 3T91, 3ZT9, 3W40), two of which have been described in published reports [93, 109]. All of these proteins conspicuously lack a third bound metal ion, in agreement with the prediction of our model. The cases of 3W40 and 3ZT9 are especially interesting and important. These recently described structures are the only ones available from members of the bacterial GN Group II set (the immediate ancestors of eukaryotic PP2C7s), the first from the prototype RsbX of *Bacillus subtilis*, the second from the RsbX homologue (“MtX”) of the bacterium *Moorella thermoacetica*. Even these structures lack a third bound metal ion, suggesting that what we have interpreted as the precursor (N) to the conserved aspartate (D) at Motif 5 is still unable to bind metals. Experimental testing of these predictions of our model of metal binding by eukaryotic PP2C7s and bacterial Group II PP2Cs will require detailed thermodynamic measurements capable of detecting possible weak third metal binding.

## Supporting Information

S1 File Combined Tabulated Supporting Information. This file contains an Excel spreadsheet with tabulated Supporting Information. Each page contains a constituent subfile. There are 14 tables (Table A–Table N). Each is cited at the appropriate place in the text. Each table contains a Legend which explains its data.
(XLSX)

S2 File Combined Graphical Supporting Information. This file contains graphical Supporting Information figures. There are 7 figures (Fig A–Fig G). Each is cited at the appropriate place in the text. Each figure contains a Legend which explains its data.
(PDF)

S3 File Original Phylogenetic Tree Output Files. This file contains the original output phylogenetic trees for the various manuscript figures, inferred by the procedures described in Materials and Methods.
(ZIP)

S4 File BEAST Log File. This file contains the combined output log information for the two BEAST runs used to infer the phylogenetic tree presented as Fig 1.
(TXT)
