# Supplementary material for: "PP2C7s", Genes Most Highly Elaborated in Photosynthetic Organisms, Reveal the Bacterial Origin and Stepwise Evolution of PPM/PP2C Protein Phosphatases
Source: PLoS One. 2015 Aug 4;10(8):e0132863. doi: 10.1371/journal.pone.0132863 (PMC4524716; doi:10.1371/journal.pone.0132863)
Supplement: S2 File — This file contains graphical Supporting Information figures. There are 7 figures (Fig A–Fig G). Each is cited at the appropriate place in the text. Each figure contains a Legend which explains its data. (PDF) [file pone.0132863.s002.pdf]

Fig A: Sequence Alignments Corresponding to Phylogenetic Trees

(Panel 1) Alignment Corresponding to Figs. 1, 2, 3

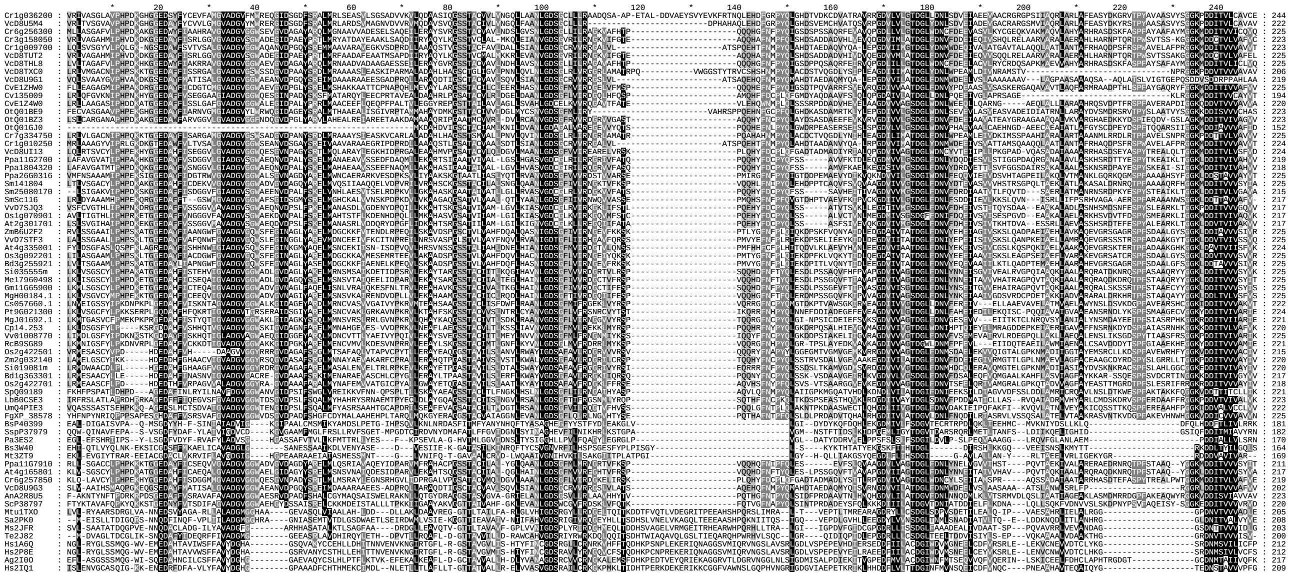

(Panel 2) Alignment Corresponding to Fig. 4

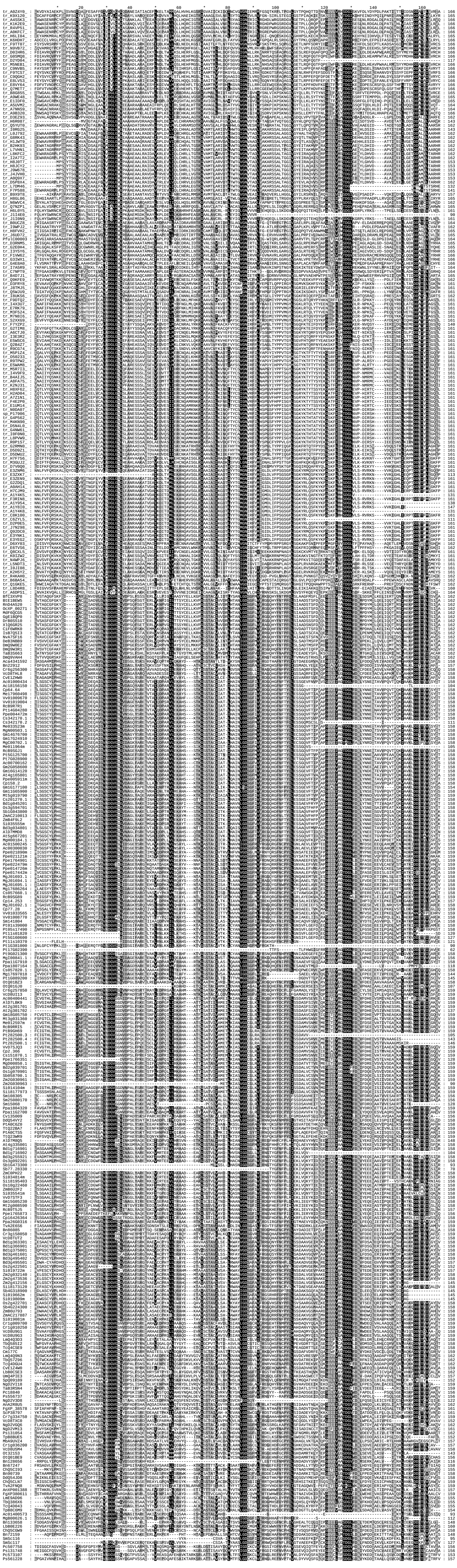

(Panel 3) Alignment Corresponding to Fig. 5

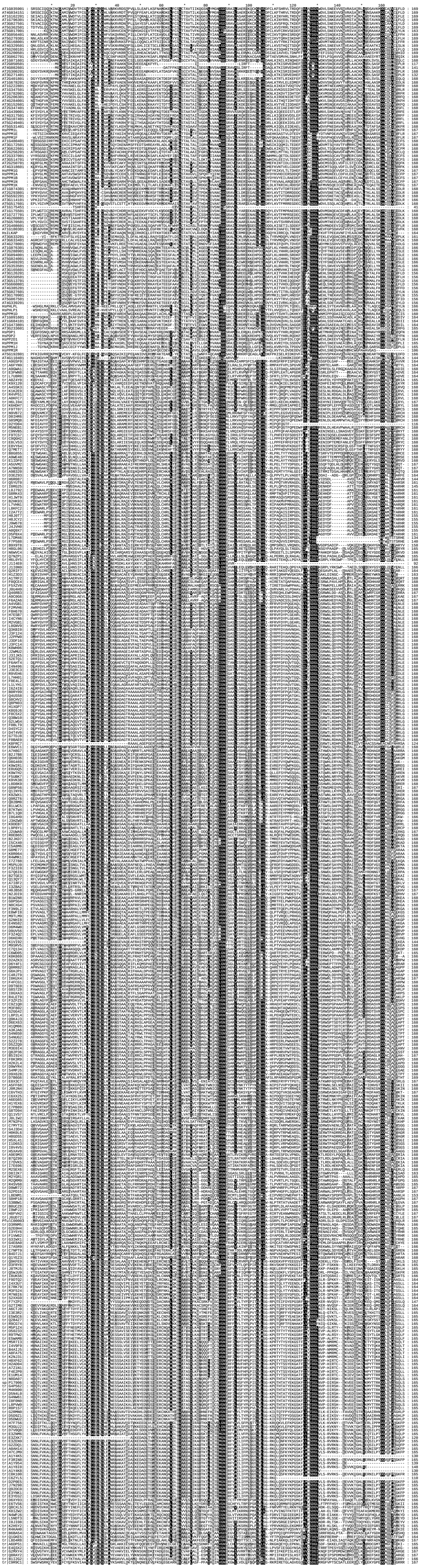

(Panel 4) Alignment Corresponding to Fig. 6

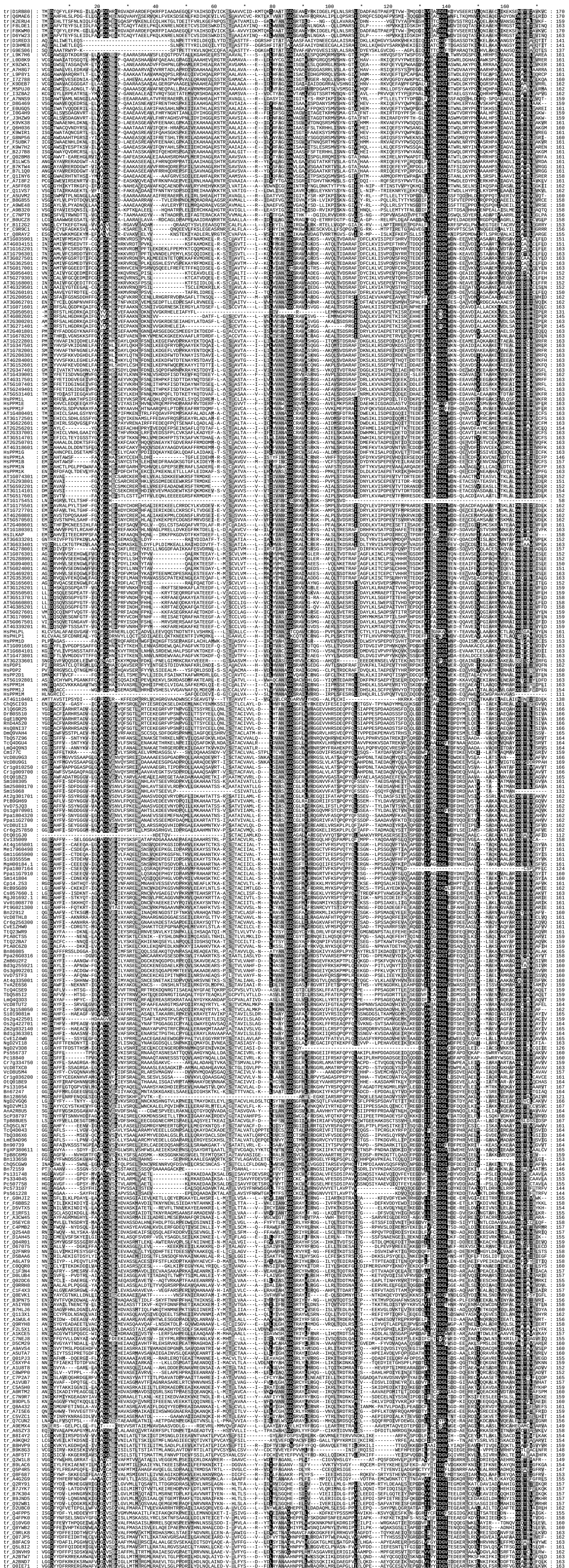

(Panel 5) Alignment Corresponding to Fig. E in S2 File

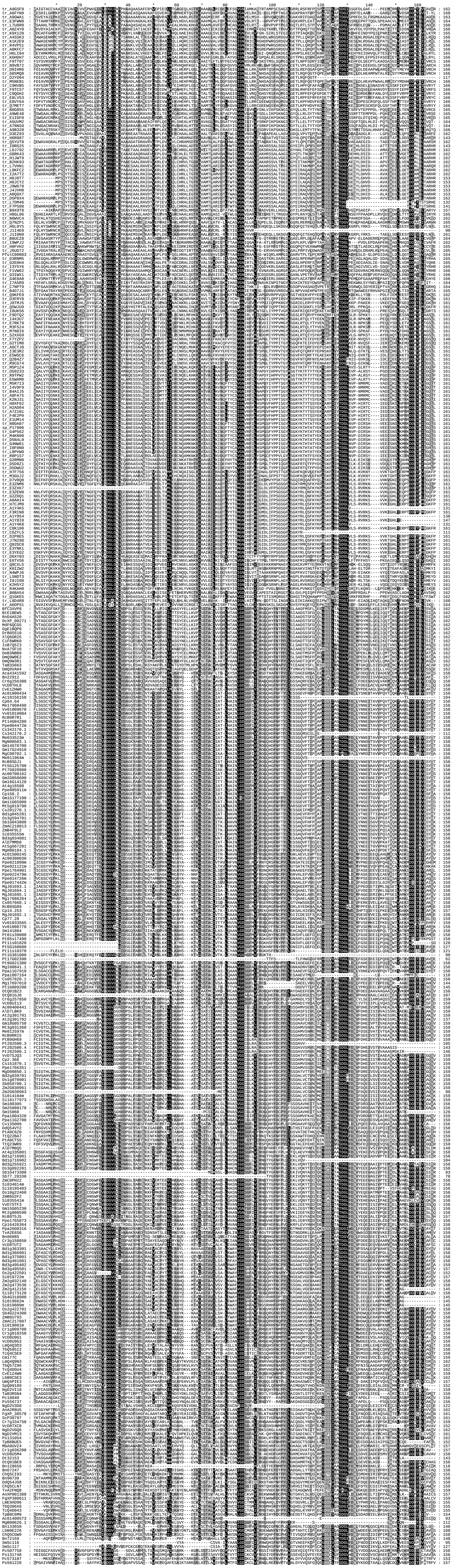

Fig A: Sequence alignments corresponding to phylogenetic trees. Candidate PP2C7 sequences were retrieved, validated, and aligned as detailed in "Materials and Methods". Phylogenetic trees were inferred as detailed in "Materials and Methods". This figure presents the sequence alignment corresponding to each phylogenetic tree presented in this report. See Table A in S1 File for a list of PP2C7 sequences.

Fig B: Diurnal gene expression data for Arabidopsis starch metabolism genes

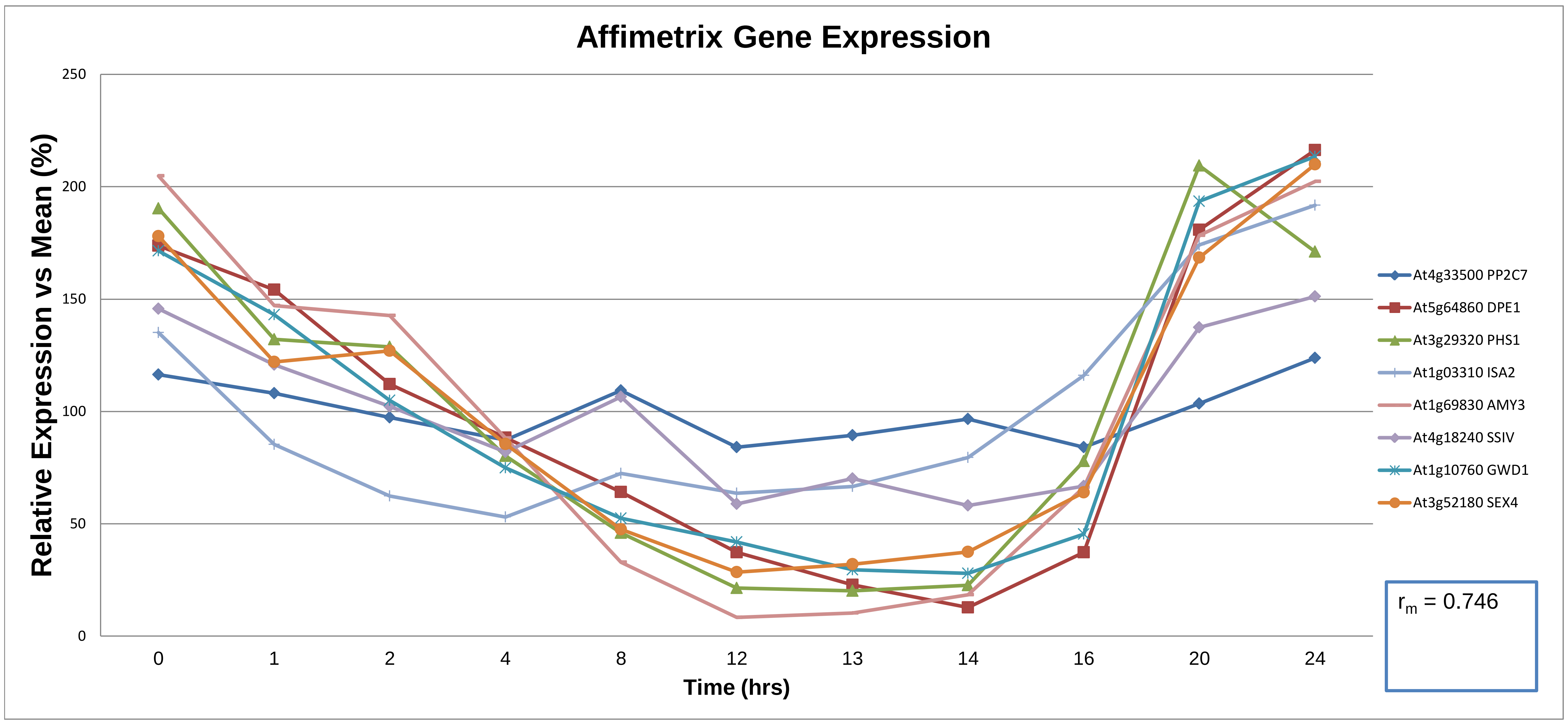

Fig B: Diurnal gene expression data for Arabidopsis starch metabolism genes. Expression data for selected Arabidopsis starch metabolic protein genes were retrieved from NCBI GEO (Gene Expression Omnibus) and placed in Microsoft Excel as detailed in Methods. Data were expressed as relative expression vs mean, using data from replicate experiments. Correlation coefficients were calculated comparing expression patterns of At4g33500 (PP2C7) to each of the seven other genes, and the mean of this set of correlation coefficients is presented in the Figure. The individual correlation coefficients are as follows: At5g64860 (DPE1 (DISPROPORTIONATING ENZYME 1)) 0.7900; At3g29320 (PHS1 (plastidial phosphorylase isozyme 1)) 0.6592; At1g03310 (ISA2 (ISOAMYLASE 2)) 0.6376; At1g69830 (AMY3 (ALPHA-AMYLASE-LIKE 3)) 0.7143; At4g18240 (SSIV (STARCH SYNTHASE 4)) 0.8896; At1g10760 (GWD1 (glucan/water dikinase 1)) 0.7751; At3g52180 (SEX4 (STARCH-EXCESS 4)) 0.7578. The first twelve hour period is the dark period, the second twelve hour period is the light period.

Fig C: Sequence Logos for Plant PP2C7 N-terminal Motifs

(Panel 1) All Plant Groups Except Dicots

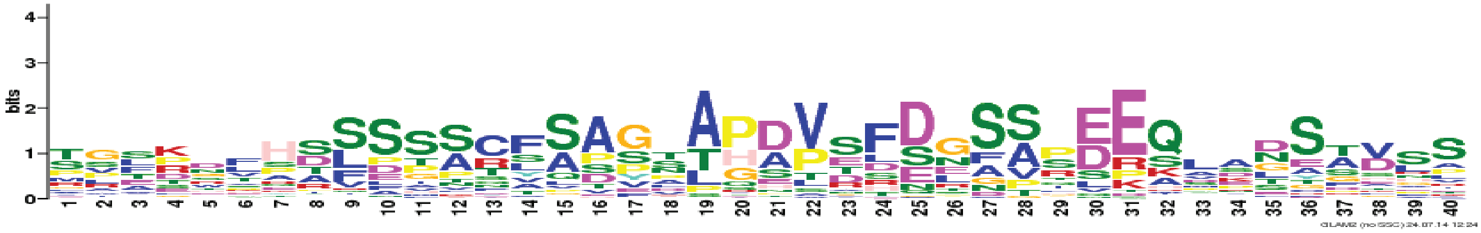

(Panel 2) At2g30170 Group

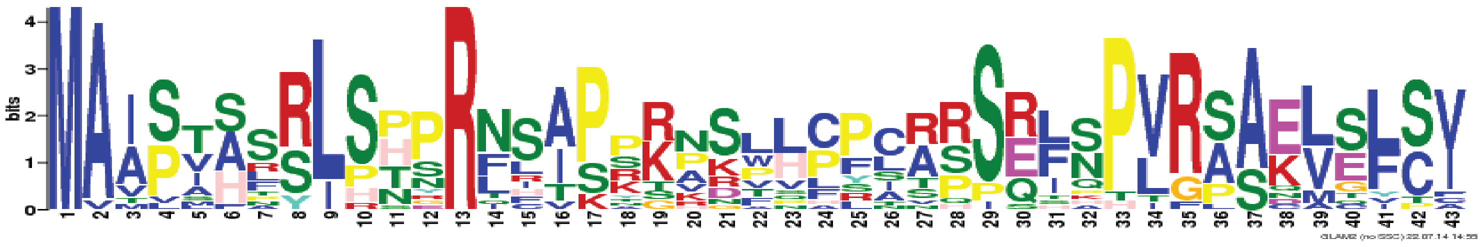

(Panel 3) At4g33500 Group

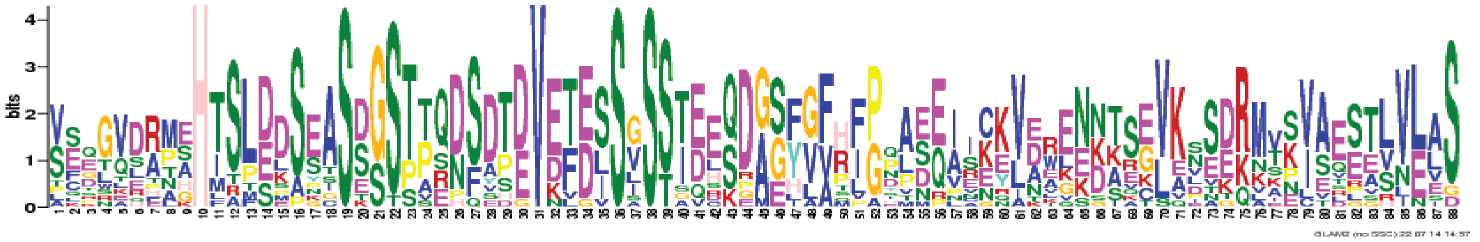

(Panel 4) At4g16580 Group

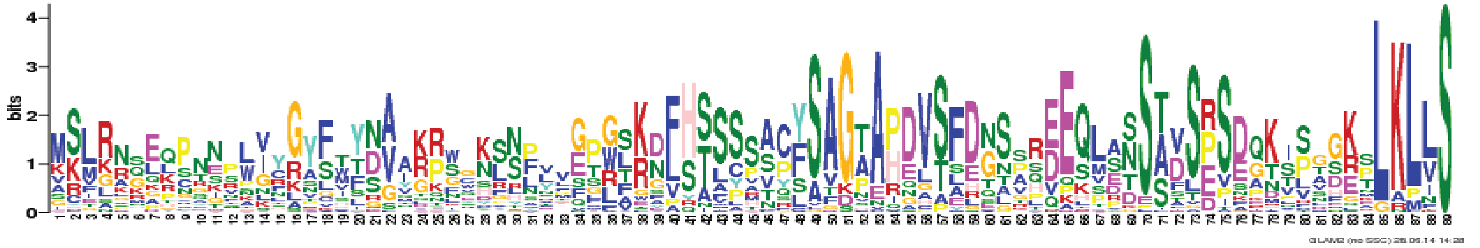

(Panel 5) Monocot Group

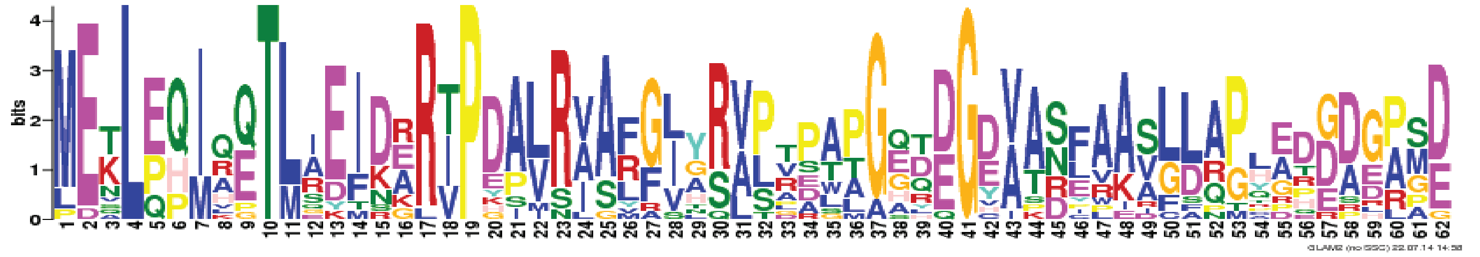

Fig C: Sequence Logos for plant N-terminal PP2C7 motifs. Sequence motifs were inferred as detailed in "Materials and Methods". Sequences are presented as logos. The corresponding sequence groups are presented in Fig. 3 (representative form) and in complete form in Table F in S1 File. Sequences shorter than 8 residues were omitted from the analysis. The composition of the analyzed sequence sets are as follows: "All Plant Sequences Except Dicots" (102 total, average length: 193.3 residues); "At2g30170 Group" (21 total, average length: 75.6 residues); "At4g33500 Group" (19 total, average length: 388.5 residues); "At4g16580 Group" (45 total, average length: 207.6 residues); "Monocot Group" (19 total, average length: 80.8 residues). The scores for the motifs are as follows: All Groups Except Dicots – 2326; At2g30170 Group – 615; At4g33500 Group – 1138; At4g16580 Group – 3941; Monocot Group – 995. The same set of N-terminal motifs is presented in conventional sequence alignment form as Fig D in S2 File. Inference of motifs in the same fashion for the "Dicot Group" yielded a result which was only 7 residues long (not shown). For this reason Dicot sequences were omitted from the "All Plant Sequences" Group.

Fig D: Conventional sequence alignments for plant PP2C7 N-terminal motifs

(Panel 1) All Groups Except Dicots

```
                      *        20         *        40         *
AlD7LBK9   : --6TRMMVPHA-RPSLRLSHPNLPNPSRV---DFLC-LCV---PSEHQPLRPELS-49 : 44
AT2G301701 : --6TRMMVPHA-IPSLRLSHPNPSRV------DFLC-RCA---PSEIQPLRPELS-46 : 41
Bd2g039701 : --5TAASRLSP-PRPRTASPPST-PP------RR-S-RFS---PLRAAKMEAVLS-43 : 39
Cs151870.1 : --1MAALALHA--SVPQSQPFIP-FP------SF-S-LST---FS-QSSNRRNLL-37 : 37
Gm15G05230 : -40VSFTHHTR-LLPNATSSPNS-DPE-----DF-DILSS---TE-HSDGSFVFR-79 : 40
Os1g070901 : --2AASSTATR-LSPPRLHAPTTPSPHLPL--RR-S-RFS---PLRAAKLEAVLT-45 : 44
Sb058700.1 : --1MAVSTASR-LSPPRFRAPPP-SLHPPT--RR-S-RFS---PVRAAKLEAVLS-43 : 43
Si014104m  : --1MAASTASR-LSTPRFLAPPA-SPHPPI--RR-S-RFS---PVRAAKLEAVLS-43 : 43
Si18177973 : --1MAASTASR-LSTPRFLAPPA-SPHPPI--RR-S-RFS---PVRAANLGTFPA-43 : 43
Zm2G030961 : -28MVASTASR-LSPPRFSTPSP-SLHPPN--RR-S-RFS---PVRAAKLEAVLS-70 : 43
Ac01400573 : 135TTTTTTAV-LEPNSVLEVNT-AI------CS-E-EDV---EE-KDRDELVSS172 : 38
AlD7M8Q5   : -48SPSKSPAS-SSSPC-PPENS-APE-----KF-D-L-V---SSTQLKDGSHVF-85 : 38
At4g335001 : -49SISPSKSS--ASSSSPPENS-APE-----KF-D-L-V---SSTQLKDGSHVF-86 : 38
Bd1g716901 : 263TSLDDSEG-SDGSTTQDSDT-DVET----ES-S-SSS---IEEQDAGYGVHV303 : 41
Mt2g008590 : -46TTTHHPHI-LPSST-SSSTP-SPQEHE--EL-DVISS---TE-RSDGSFVFT-87 : 42
MgB00626.1 : 432TVEESKKD--DGAESLTVDL-SVGPEITEAD-D-EAP---SDDSIEESLHEV475 : 44
Os10g22460 : -29SPSPRHHR-RPHSTATACRA-AP------DL---HSS---TE-LADGSIVFR-65 : 37
Os3g092201 : 279TSLDDSEA-SDGSTTQDFDT-DVET----ES-S-GSS---IEEQDMGYGVHI319 : 41
Ppa26G0316 : 113SSETNIAE-VNSSDSGDVKA-SVNI----SR-N-ESVV--GN-SAAGAEDSS153 : 41
RcB9T5J5   : -29SSSKRTSC-FSFKTVSTPKT-TT------SS-S-S-S---SS-SLCSSRDSV-65 : 37
Si035541m  : 142GLEESEAA-SEGSTVQDFDT-DVET----ES-S-GSS---GDEQGAEFGVPL182 : 41
Si034814m  : 273MSLDDSEA-SDGSTTQDSDT-DVET----ES-S-GSS---IEEQDAGYGAHI313 : 41
SmSc116    : -73LPFDEIAS-QFSTRASEETT-HV------TF-D-ESAVSLPQPAKKFSTHNS114 : 42
VvD7STF3   : -37RPPSHFSS-FLSYCSSTPTPSHS------DD-D-EFG---LL-SSTDCSDGS-75 : 39
ZmC0PH22   : 277TSLDDSEA-SDGSTTQDSDT-DVET----ES-S-VSS---IEEQEAGYGAHI317 : 41
ZmB6U2F2   : 241GLEDSEAA-SEGSTAQDFYT-DVET----ES-S-GSS---SDEQRAEFGFSL281 : 41
Ac01000434 : 212GRSKDFHS-VSSAFYSTGVAPEV------SF-D-GSP---HEESLRSSADSS251 : 40
Ac00700162 : 127TVSKDFHTCSSSSSSGSYVTGAAPDAP--SH---GSTQE-EVEQLETSSASE172 : 46
AlD7MCE3   : 168MGFRGLHS-SLSNRLSAGNAPDV------SL-D-NSV---TEEQVRDSSDSV207 : 40
AlD7MMD8   : 126TLTKSVHT-SPMACFSVGPAHELS-----SL-N-GGS---QE-SPPTTTSLK165 : 40
AT4G16580  : 168MGFRGLHS-SLSNRLSAGNAPDV------SL-D-NSV---TDEQVRDSSDSV207 : 40
AT5G667201 : 126TLSKSVHT-SPMACFSVGPAHELS-----SL-N-GGS---QE-SPPTTTTSL165 : 40
AT5G667202 : 126TLSKSVHT-SPMACFSVGPAHELS-----SL-N-GGS---QE-SPPTTTTSL165 : 40
Bd1g045201 : 176SVEPWTRD-LSTSCVAPYSAGATERQH--TL-D-EAV---QDKQMDTASDGK219 : 44
Bd1g045202 : -55SVEPWTRD-LSTSCVAPYSAGATERQH--TL-D-EAV---QDKQMDTASDGK-98 : 44
Bd3g034801 : -84GGVRSFAT-GAAPEHVSFSA-AV------RE-E-GDSQ--SEKPAVTSDKNM123 : 40
Cp158.2    : 157PFLKNVHT-FSSVCFSAGPVHDVS-----CD-D-N-S---QEQELEDSPISS196 : 40
Cp64.64    : 190SGLRYFHI-SSSTCFSAGTAPDV------SF-D-NSA---REEQVASSIESS229 : 40
Cs201270.1 : 153VGVTNLHA-LPHACYAAGTANSP------AF-D-SNS---RDDQFPNSTTLP192 : 40
Cs342170.1 : 206PGVKDLHS-SSTSQFAAGSAPNV------SF-D-NSA---REEQLANSTDSS245 : 40
Cs342170.2 : 206PGVKDLHS-SSTSQFAAGSAPNV------SF-D-NSA---REEQLANSTDSS245 : 40
Gm1G177100 : 108LRTLWV-T-LEARFGGDGAAHAV------SF-D-GSP---PDEQLANSFFSP146 : 39
Gm2G058600 : -70PSSLYKNS-SFFARCSAETTPHVQHL---AT-S-TFS---ID-QTNFGGERL111 : 42
Gm11G65900 : -75SWLKNFSA-SSSACYSAGAAHAV------SF-D-GSP---PDEQLANSSFSP114 : 40
Gm14G76700 : 209SGSGDFHT-LSSSCYSVGPAHDV------PF-D-TSA---HEEQLSSSADPS248 : 40
Gm16G14120 : -69PSSLHRNS--SFACCSAETTPHVQHL---AT-S-TFS---ID-QTNFGGERL109 : 41
Gm17G24910 : 209SGSGDFHT-LSSSCYSVGPAHDV------PF-D-TAA---REEQLSSSADSS248 : 40
Mt5g019790 : 152PWTKNFSA-SYSACCLAGAAHDL------SF-D-TSP---PDEKLENSSTLA191 : 40
Me17960498 : --8YWFGPKPR-LAIAD-PAGIAPDV------TF-E-NSA---RDEQLENSAGSS-46 : 39
Me011964m  : 126PLLKNFHT-MPSLQFSAGAARDV------SF-E-GNS---REEQLANSTVVS165 : 40
Me033523m  : 212TGSRYFHS-SSPSYLSAGTAPDV------TF-E-NSA---CEERLENSSVSS251 : 40
MgN00503.1 : 207FGFEGFHN-ISPSYSSASTAADV------TF-D-N-PI--KEEQHSNSADSS246 : 40
MgH00184.1 : 128KGMSEPRS-FSSSCYSDGNAPEVS-----SDGS-LSVE--TLSSLALSTDES170 : 43
MgC00702.1 : 202LGFEGLHI-SSSTCSSAGTARDV------SF-G-NSA---REEHESSSADES241 : 40
Os3g594701 : 178SVEPWLRD-FSTSCVAPYSAGATEHQL--SL-D-EAV---QDKQMDNSTVGP221 : 44
Ppa11G7910 : -61SRHPFVPS-FFSKFFSSSRTPSA------DH-N-PASFD-SSKQQALTASEA102 : 42
Ppe005911m : 141PWLRNFHN-SSSVCCAAGAAHNV------SF-D-GSS---SDEQLANSTILS180 : 40
Ppe004472m : 212SGSREFHS--SSTCLSAGTAHDV------SF-D-NSA---PEEQLSSSADSS250 : 39
Pt5G125700 : 126TLLKNLYS-SSSVCFSAGAAQDV------SF-D-GNS---RKEQVVDSTVVS165 : 40
Pt7G028900 : 126PLLKNLYS-SSSVCVFGRAAQDV------SF-D-GNS---SEEQSVDSTVVS165 : 40
Pt14G04280 : 214TGFRDFQS-SAHSCFAAGTAPDV------TY-E-NST---REEQPEGSASSE253 : 40
Pt14G04280 : 214TGFRDFQS-SAHSCFAAGTAPDV------TY-E-NST---REEQPEGSASSE253 : 40
RcB9SGJ1   : 126PLLKNLHT-LSSMQFSAGAAPDV------SF-D-GNP---HEEQLTNSMVSS165 : 40
RcB9R7R1   : 215SGARFLHS-SSPACLSAGTAPDV------TF-E-NSG---REEQLETSTVSS254 : 40
Sb1G043800 : 173AVEPWTKD-FSTSCAAPYSAGAT------EH-Q-L-S---LDEKMDNSSVAS211 : 39
Si035555m  : 172AVEPWARD-FSSSCVAPYSTGAT------EH-Q-L-S---LDEKMDDSTVTS210 : 39
Sm141804   : -63RGTRRVPP-SSSSSSSSSSA-DSS-----NF-E-EGA---PL-SVSEAFSRA101 : 39
Vv01019004 : -35PWFKNFHT-WSSSCYSAGAAPDV------SF-G-GSS---SDEQLSKSAASS-74 : 40
ZmB4F9L2   : 173TVEPWTKD-FSTACAAPYSAGAT------ED-Q-L-P---LNEKMNSSTVGM211 : 39
ZmAC210013 : 107AVEPWTKG-FSTSCAAPYSAGAT------EH-Q-L-S---LDEKVDNSTVAS145 : 39
Sb4G224300 : -17EIKERTPD-VSISRFVASVLDDSP-----GT-E-H-A---PSEWKIGDDDDA-56 : 40
Sb4G318900 : -35TPAPGEYD--EVADFAASLLQTPPP----TE-D-GDG---PD-ADADDRGRT-74 : 40
Si019662m  : -35TPAPGEDD--EVADFAASLLQPPA-----TD-D-G-A---GD-RGPTEHDAA-72 : 38
Si019722m  : -31TPAPGHHD--EVANFAAVLL-AP------RD---G-A---SEPMDCDEPVAP-66 : 36
Si18173120 : -62REIDERPD--EIANFAAVLL-PPRAHG--EH-D-GDAC--PQ-WDDDEDVPR103 : 42
Zm2g032140 : 212RPTTGQQD--DAANLAAVLL-APL-----DD-D-G-A---SDPKAIDCDKNP249 : 38
ZmB6U793   : -19EIKERTPD-VNISRFVASVLGDWM-----ET-E-YAT---SERKRGDAGHGR-59 : 41
Zm2g412156 : -31RPTTGQQD--DAANLAAVLL-APL-----DD-D-G-A---SDPKAIDCDKNP-68 : 38
Zm2g473536 : -31RRTTGQQD--DAANLAAVLL-APL-----DD-D-G-A---SDPITSDCDKNP-68 : 38
ZmAC217887 : -13EINERTPH-VSISRFVASVLVEWL-----QT-E-YAT---SERKSGDAAGHE-53 : 41
```

(Panel 2) At2g30170 Group

```
                      *         20         *         40         *         60         *         80
Ac00400441 : -1MAISMPK---------------SSSISPSNQFLQSF--SRN-TKKKNSVVC-CNQS-ELSPT-----RL-ELS---ICV50 : 50
AlD7LBK9   : -1MAIPVTRMMVPHARP-------SLRLSHPNL-------P-N--PSRVDFLCLCVPS-EHQPL-----RP-ELS---LSV52 : 52
AT2G301701 : -1MAIPVTRMMVPHAIP-------SLRLSH----------P-N--PSRVDFLCRCAPS-EIQPL-----RP-ELS---LSV49 : 49
Bd2g039701 : -1MAASTA----------------ASRLSPPR--------PRT-ASP-PSTPP--RRS-RFSPL-----RAAKMEAV-LSV44 : 44
Cp2.366    : -1MAVPVFRIGIPESQQLCAAHLHSPRLPN----------G-NSIPR-KSTQLFCTSSQELNPVRCS--RS-QLS---FCV61 : 61
Cs151870.1 : -1MAALALHASVPQSQPFIPFP--SFSLSTF---------S-QS-SNRRNLLF-CAPP-QLHHV-----RS-EMT---LSV55 : 55
Me012597m  : -1MAIPVLWDSIFRTYPLF-----HFSIRNLS--------NRNSIPRKNKWLC-LASP-QLNPV-----GS-EVS---FCV55 : 55
MgD00850.1 : -1MAIPLFTTISSYSTTTTTHR--HRYLHH----------P-IF-SY-NKLLC-CASS-SSPPVSSNFVGSSEAGVC-LSI61 : 61
Mt3g031360 : -1MAISILRAVMVSNSHCQSQPLIH-YISSID--------E-N-AKRRKRVVFSSSHSSELNPVI----RSSEVS---FSF61 : 61
Os1g070901 : -1MAASST----------------ATRLSP----------PRLHAPTTPSPHLPLRRS-RFSPL-----RAAKLEAV-LTI46 : 46
Ppa1804320 : -1MATPAIAVRP------------HARLHHHHQQLVGP--SRLCAPPGNGWNH-LSRI-RIAPV-----FP-CVQFHSYSC57 : 57
PtB9GH69   : -1MAMVTVFRASI-----------SRSLPTSFPNLLSSSSNRNSIPKKHRLLCYAASS-QTKTI-----RS-EVS---FCI58 : 58
RcB9RRI5   : -1MAISISRCNQFF----------HSSLPNLP--------YRNSIPKKNKWLCFATSS-QLNPV-----GS-DVC---VCV51 : 51
Sb058700.1 : -1MAVST-----------------ASRLSP----------PRFRAPP-PSLHPPTRRS-RFSPV-----RAAKLEAV-LSI44 : 44
Si014104m  : -1MAAST-----------------ASRLST----------PRFLAPP-ASPHPPIRRS-RFSPV-----RAAKLEAV-LSI44 : 44
Si18177973 : -1MAAST-----------------ASRLST----------PRFLAPP-ASPHPPIRRS-RFSPV-----RAANLGT--FPA43 : 43
VvD7SJQ3   : -1MAIPILKAAISDSHEF------FNSLSHTTRLLSIPKKRRLIVSASASASA-SAPS-EINPL-----RS-EVS---FCV62 : 62
Zm2G030961 : 28MVAST-----------------ASRLSP----------PRFSTPS-PSLHPPNRRS-RFSPV-----RAAKLEAV-LSI71 : 44
```

(Panel 3) At4g33500 Group

```
                                 *         20         *         40         *         60         *         80         *         100
Ac01400573 : -41SESSKQRLAHL--M-MAK--ISK-S--PT-NSSSE-NFDI-VSTT--VSSDGSFVFMFGNAMELNKK-EVV-DSEEL-QVSDKKKNVETSSLNEID120 : 80
AlD7M8Q5   : -38SSQLQLANS-IS-PSKSP--ASSSSPCPPENSAPE-KFDL-VSST--QLKDGSHVFRFGDASEIEKYLEAQEKARCV-ELEKQNAKIAEEAS-ELS123 : 86
At4g335001 : -41SSQLQLANS-IS-PSKSS--ASSSS--PPENSAPE-KFDL-VSST--QLKDGSHVFRFGDASEIEKYLEAEEKARCV-EVETQNAKIAEEAS-EVS124 : 84
Bd1g716901 : 254VQEGVDRME-TS-LDDSE--GSDGS--TTQDSDTDVETESSSSSI--EEQDAGYGVHVPPMEQPICEVTRESNISEV-KSSDRMVSVAVSTHVLAS340 : 87
Gm15G05230 : -36PKPWVSFTHHTR-L-LPN--A-T-S--SP-NSDPE-DFDI-LSST--EHSDGSFVFRFASANEIREQLDELNKKKKSDALNKKKKLAREGVLEEG118 : 83
Mt2g008590 : -44TFTTTHHPH-I--L-PSS--TSS-S--TPSPQEHE-ELDV-ISST--ERSDGSFVFTFGNASEIREKIAEL-NKQKL-VPEG-VVE-EEGVSVLVS122 : 79
Os10g22460 : 132ASGGAEATA-TSGLEDAGEEASDGS--TARDSDTDVDTESSASTAADDDQPAEFAVPPPPAEEVCNKVDWEKDTSEV-KNTDRMVPVASSTLVLAS223 : 92
Os3g092201 : 270VEEGVDRME-TS-LDDSE--ASDGS--TTQDFDTDVETESSGSSI--EEQDMGYGVHIPHTEQAICEVARGNKSSEV-KSSDRMSSVTLPTLILAS356 : 87
RcB9T5J5   : -38SFKTVSTPK-TT-T-SSS--SSSSSLCSSRDSVSG-DVDI-ISTT--EHSDGSLLFQFGELNEISENVKIGESKVTL-KDAE-LEN-ADEEVNNLS121 : 84
Si035541m  : 131LEQREEAAG-TAGLEESEA-ASEGS--TVQDFDTDVETESSGSSG--DEQGAEFGVPLPIVERNSKEVDWKKDTSEV-KDSDRMVEIAQSELVLLS219 : 89
Si034814m  : 264VCDGVDRME-MS-LDDSE--ASDGS--TTQDSDTDVETESSGSSI--EEQDAGYGAHIPQLDQAICKVNRENNTSGV-KNSERMTSVSESTLVLAS350 : 87
VvD7STF3   : -32VLRNQRPPSHFSSF-LSY--CSS-TP-TPSHSDDD-EFGL-LSST--DCSDGSILFRFGYASEIAGRVELE-GAEGL-AEVEKASKCVESEVVEES116 : 85
ZmC0PH22   : 268SEEGVDRMG-TS-LDDSE--ASDGS--TTQDSDTDVETESSVSSI--EEQEAGYGAHIPQPDPAVCKVAKENNTAGV-KISDRMTSVSELTLVLAS354 : 87
ZmB6U2F2   : 230VSGGLDEAA-TSGLEDSEA-ASEGS--TAQDFYTDVETESSGSSS--DEQRAEFGFSLPPPEQVSNKADWKNDTSEV-KSSDRMIPIAQRTRVLSS318 : 89
```

(Panel 4) At4g16580 Group

```
                           *         20         *         40         *         60         *         80         *         100         *
Ac01000434  : 180ASLLNQGPS-NNRLVH-GYSFY-D-QTK-VNRNL-----NPFGRSKDFHSVSSAFYSTG----VAPEVSFDGSPHEE---SLRSSAD-S-SDQKVVADRSLKLLS265
Ac00700162  : -92MSLRDSEPSSNTNLVI-GYFMF-DAIRKAFNYNP-----LQGTVSKDFHTCSSSSSGSYVTGAAPDAPSHGSTQEEVE-QLETSSA-SE-DKNSLSNRTLKLLS186
AlD7MCE3    : 133MRLRGKDHQ-EKSTIY-AYFAY-RGAKRWIYLNQQ----RRGMGFRGLHSSLSNRLSAG----NAPDVSLDNSVTEE---QVRDSSD-S-VADKLCT-KPLKLVS220
AlD7MMD8    : -92ASMRLGKRV-GITKNR-LVCHY-SAIELLEKSRA-----LFGTLTKSVHTSPMACFSVG----PAHELSSLNGGSQE---SPPT-T--------TSL-KSLRLVS171
AT4G16580   : 133MRLRGKDHN-EKSTIC-AYFAY-RGAKRWIYLNQQ----RRGMGFRGLHSSLSNRLSAG----NAPDVSLDNSVTDE---QVRDSSD-S-VAAKLCT-KPLKLVS220
AT5G667201  : -92ASMRLGKRG-GMMKNR-LVCHY-SVVDPLEKSRA-----LFGTLSKSVHTSPMACFSVG----PAHELSSLNGGSQE---SPPT-T-------TTSL-KSLRLVS172
AT5G667202  : -92ASMRLGKRG-GMMKNR-LVCHY-SVVDPLEKSRA-----LFGTLSKSVHTSPMACFSVG----PAHELSSLNGGSQE---SPPT-T--------T-T--SLKLVS169
Bd1g045201  : 144KKVGANLKC-REQWGSARTFWT-SAAGPGSKLSF-----SVEPWTRDLSTSCVAPYSAGA---TERQHTLDEAVQDK---QMDT-A----SDGKSPASKALKLLS230
Bd1g045202  : -23KKVGANLKC-REQWGSARTFWT-SAAGPGSKLSF-----SVEPWTRDLSTSCVAPYSAGA---TERQHTLDEAVQDK---QMDT-A----SDGKSPASKALKLLS109
Bd3g034801  : -50GPAAGGGGG-AGSWWF-RCAVS-SVPRP-G-L-L-----VEQLLVGGVRSF-ATG-AA-----PEH-VSFSAAVREEGDSQSEKPAV-T-SDKNMLGDRSLKLLS133
Cp158.2     : 124ISFRKQEHY-NGHLVC-GYFMP-KVQGN-TSSNT-----IFGPFLKNVHTFSSVCFSAG----PVHDVSCDDNSQEQ---ELED-S------P-ISSSQPLNLLS204
Cp64.64     : 156MSLKSQEQP-GNYSVY-GYLMY-NAAKRWVNVWP-----YTESGLRYFHISSSTCFSAG----TAPDVSFDNSAREE---QVASSIE-S-SEQKIPAGKTLKLLS243
Cs201270.1  : 122MSLRRKQAS-NNCLI--SNSSI-D-LMR-VKGNC-----FLQVGVTNLHALPHACYAAG----TANSPAFDSNSRDD---QFPNSTT-LPSKG-LLGERTLKLLS206
Cs342170.1  : 172MSLKNQEQP-NNNGIY-GYVTY-NVAKRFCSSYL-----NTVPGVKDLHSSSTSQFAAG----SAPNVSFDNSAREE---QLANSTD-S-SEQKISMGKSLKLVS259
Cs342170.2  : 172MSLKNQEQP-NNNGIY-GYVTY-NVAKRFCSSYL-----NTVPGVKDLHSSSTSQFAAG----SAPNVSFDNSAREE---QLANSTD-S-SEQKISMGKSLKLVS259
Gm1G177100  : -71SCFANSEML-TKKDSL-LN-MF-Q-NRPFMKISFQKKPSLVNLRTLWV-TL-EARFGGDG---AAHAVSFDGSPPDE---QLANSFF-S-PDPIIVGGKPLKMLS160
Gm2G058600  : -30ERTRRVALR-ANVNLR-KPKPLNGGILNFGCSTS-----DASWRSWNPSSLYKNS-SF-----FAR-CSAETTPHVQ---HLATSTF-S-IDQTNFGGERLKLFS115
Gm11G65900  : -41VNLRRLQPL-YGPLSF-GCSTF-DANRRIRDSSL-----LHGSWLKNFSASSSACYSAG----AAHAVSFDGSPPDE---QLANSSF-S-PDPTIVGGKPLKMLS128
Gm14G76700  : 175MSLRNHQQP-DNSAVY-GYFIY-NAAKTWCNSHP-----YMQSGSGDFHTLSSSCYSVG----PAHDVPFDTSAHEE---QLSSSAD--PSEQKTPSGKTLKLIS262
Gm16G14120  : -30GETRRGALR-ATVNLR-KPKTL-SGILNFGCSTS-----DASWRSWNPSSLHRNS-SF-----AC--CSAETTPHVQ---HLATSTF-S-IDQTNFGGERLKLFS113
Gm17G24910  : 175MRLKNHQQP-DNTAIY-GYLIY-NAAKTWCNSHP-----YMQSGSGDFHTLSSSCYSVG----PAHDVPFDTAAREE---QLSSSAD-S-SEQKTPLGKTLKLIS262
Mt5g019790  : 117LSLRRPQQVLYGPLNF-GRSTF-DASWRIQNSGL-----LHGPWTKNFSASYSACCLAG----AAHDLSFDTSPPDE---KLENSST-L-ANITTLDRKPLKMLS205
Me011964m   : -92MRMRKGELP-NSRLVC-GYSSF-DAIRSGEMNNF-----VFGPLLKNFHTMPSLQFSAG----AARDVSFEGNSREE---QLANSTV-------VSG-LNLKLLS173
Me033523m   : 181MSLNNRKQP-TNSPIY-GYFIY-NVMKK-W-F-P-----YIETGSRYFHSSSPSYLSAG----TAPDVTFENSACEE---RLENSSV-S-SEQRHSAGKMLKLIS265
MgN00503.1  : 173MSLSRKEQP-NNFLLY-GYFVY-NVVKRKGNGNP-----LLGFGFEGFHNISPSYSSAS----TAADVTFDNPIKEE---QHSNSAD-S-SNLNIPIDRTLKLKS260
MgH00184.1  : -92VSIRSG-GN-GSPNGF-RESPIFDFVRR-CSSNSGGA--RSSKGMSEPRSFSSSCYSDG----NAPEVSSDGSLSVE---TLSSLAL-S-TDE-SRNHSVLKLVS180
MgC00702.1  : 168MSLGNKEQP-NNFLLY-GFFMY-NVAKRKGNGNP-----FVGLGFEGLHISSSTCSSAG----TARDVSFGNSAREE---HESSSAD--ESKQNAPTDRSLKLNS255
Os3g594701  : 146KKVGVSLKC-REPWGPSRAFWT-NAIGPSYKLSF-----SVEPWLRDFSTSCVAPYSAGA---TEHQLSLDEAVQDK---QMDNSTV-G-PDGKPRAPGPLKLVS235
Ppa11G7910  : -35GKGSPSARN-TKPSVT-GSSTTPGGYQPSRHP-F-----VPSFFSKFF-SSSRTP-SAD----HNP-ASFDSSKQQA---LTASEAV-SRVFATPSVHGPL-LNS119
Ppe005911m  : 107VYFNDR-RQ-SSFLK--ASLSF-R-KKESYNSHLISV--HVGPWLRNFHNSSSVCCAAG----AAHNVSFDGSSSDE---QLANSTI-L-SDPPILGEKALKLLS194
Ppe004472m  : 178MSLKKRGLS-NTNAIF-GYFIY-EVGKRWSNSSP-----TKGSGSREFHSS-STCLSAG----TAHDVSFDNSAPEE---QLSSSAD-S-SDRKVTDGKSLKLTS264
Pt5G125700  : -91VKMRNRELP-NSRLVC-GNFML-DAMRGNGKANNL----VVGTLLKNLYSSSSVCFSAG----AAQDVSFDGNSRKE---QVVDSTV-V-SGQNTVDDRNLKLLS179
Pt7G028900  : -91VKMRNGELP-KSRLVC-GNLML-DAIRRNGKANNL----VLGPLLKNLYSSSSVC-VFGR---AAQDVSFDGNSSEE---QSVDSTV-------VSG-LNLKLLS173
Pt14G04280  : 180MSLKNQEQP-TNSPIY-GYFVY-NVAKRWCDFSP-----YMETGFRDFQSSAHSCFAAG----TAPDVTYENSTREE---QPEGSA--S-SEQKISTGKMLKLLS266
Pt14G04280  : 180MSLKNQEQP-TNSPIY-GYFVY-NVAKRWCDFSP-----YMETGFRDFQSSAHSCFAAG----TAPDVTYENSTREE---QPEGSA--S-SEQKISTGKMLKLLS266
RcB9SGJ1    : -91IRMRNGELP-NSRLVC-GYSSF-DAIRRTGQLNYF----GVGPLLKNLHTLSSMQFSAG----AAPDVSFDGNPHEE---QLTN-S-------MVSS-QTLKLLS172
RcB9R7R1    : 181MSLKKEEQS-SNFPIY-GYFVC-NVMKKWYSFSS-----YIESGARFLHSSSPACLSAG----TAPDVTFENSGREE---QLETSTV-S-SEEKISSGKILKLIS268
Sb1G043800  : 142KKIGVSLKC-REPWGN-RAFWT-NAAGPGWKLSF-----AVEPWTKDFSTSCAAPYSAG-----A---TEHQLSLDE---KMDNSSV-A-SDGKSPVSEKLKLLS225
Si035555m   : 141KKFGASLKC-CEPWGN-RSFWT-NAVGPSWKLSF-----AVEPWARDFSSSCVAPYSTG-----A---TEHQLSLDE---KMDDSTV-T-SDGKAPTSESLKLVS224
Sm141804    : --6SCELRMPTP-QIVWKL-KAGTS-SSYRQSQHRLN-----VLFGRAKAA-AAYPVYSSAK----TAIGGAARGTRRVPP--SSSSSS-S-SSADSSNFEEGAPLS-93
Vv01019004  : --1MSLRNQEPP-NRSLVC-GYLIF-DVTRRNCISNP-----LDGPWFKNFHTWSSSCYSAG----AAPDVSFGGSSSDE---QLSKSAA-S-SDQAILGHRTLKLIS-88
ZmB4F9L2    : 142KKIGASLKC-REPWGN-SAFWT-NATGPGWKLSF-----TVEPWTKDFSTACAAPYSAG-----A---TEDQLPLNE---KMNSSTV-----GMSPVSEKLKLLS222
ZmAC210013  : -76KKVAASLKS-SEPWGN-RAFWT-NAAGPGWKLSF-----AVEPWTKGFSTSCAAPYSAG-----A---TEHQLSLDE---KVDNSTVASDSDGKSPVSEKLKFLS161
```

(Panel 5) Monocot Group

```
                         *        20         *        40         *        60         *
Bd1g366901 : --1MDSLPQ---IRQTLEKIRARTPEPLRLAFRIHF-GSL-PASAAGARQ-DVARYLAALS---NMYEPE-DL-ME-62 : 62
Bd1g375001 : --1MEVLPPAE-ILPTLAEMKARTPKPMRIAYAIRY-GRL-PASAAAGRE-DVARCVAALA---RTYEPDMDDLME-66 : 66
Bd3g495501 : --1METLEP---IQQTLREIDKRVPYSLRAAFGLA--HR--PVALP-SDDGDIASFVASFF---QPQDDG-PGPAE-61 : 61
Os2g422501 : --5METLEQ---IKETLRETSKLVPDIVRAAVGLEHHYQ--TVELP-HDDGCVKSFAAAFL---RPQAQEQAH-GD-67 : 63
Os2g422701 : --1MEALPQ---IRQTLSEIDRRIPDALRVAMGLR--LR--PTAGA-ALE-EVTRIAASCLP--RPCPEGGDDPME-62 : 62
Sb4G224300 : --5LEKLEQ---IQGTLAEIKERTPD-VSISRFV---ASV-LDDSP-GTE-HAPSEWKIGDDDDAGAGHG-RRRAE-66 : 62
Sb4G318900 : --1MEKLQQ---IRQTLQYIDARVPDALRVALGLG--NRVSPTPAPGEYD-EVADFAASLLQTPPPTEDG-DG-PD-65 : 65
Si019081m  : --5PETLEP---IQETLREINERTPE-VRVGRFS---TRV-LL-AL-GTE-VASSPPEDGD---AGHRHR-AERPD-62 : 58
Si019662m  : --1MENLEQ---IHQTLGDIDARVPDALRVALGLG--YRASPTPAPGEDD-EVADFAASLL---QPPATD-DGAGD-63 : 63
Si019809m  : --1MENLEQ---IHQTLSDIDGRIPDAIRVALGL---HRVSPPPAPGEDD-DVADFAISLL---QPLATE-DG-GD-61 : 61
Si019722m  : --1MEQ--H---MVQTLIEIDGRIPGALRVAFGIS--GR--PTPAPGHHD-EVANFAAVLL---APR-DGASEPMD-59 : 59
Zm2g032140 : 182MEK--H---MAETMIEFDRRIPDALRAAFGI---YRA-SRPTTGQQD-DAANLAAVLL---APL-DD-DGASD239 : 58
ZmB6U793   : --5LETL-QLQQIQQTLAEIKERTPD-VNISRFV---ASV-LGDWM-ETE-YATSERKRGD---AGHGRG-AGPSD-65 : 61
Zm2g412156 : --1MEK--H---MAETMIEFDRRIPDALRAAFGI---YRA-SRPTTGQQD-DAANLAAVLL---APL-DD-DGASD-58 : 58
Zm2g473536 : --1MEK--H---MAETMIEFDRRIPDALRAAFGI---YRA-SRRTTGQQD-DAANLAAVLL---APL-DD-DGASD-58 : 58
ZmAC217887 : --1METLQQ---IQETLAEINERTPH-VSISRFV---ASV-LVEWL-QTE-YATSERKSGDA--AGHERD-AEPSG-60 : 60
```

Fig D: Conventional Sequence Alignments for plant N-terminal PP2C7 motifs.
Sequence motifs were inferred as detailed in “Materials and Methods”. Sequences are presented as conventional alignments. The corresponding sequence logos are presented in Fig C in S2 File. The corresponding sequence groups are presented in Fig. 3 (representative form) and in complete form in Table F in S1 File. Details of the composition and characteristics of each of the sequence groups used for motif inference are presented in the legend to Fig C in S2 File.

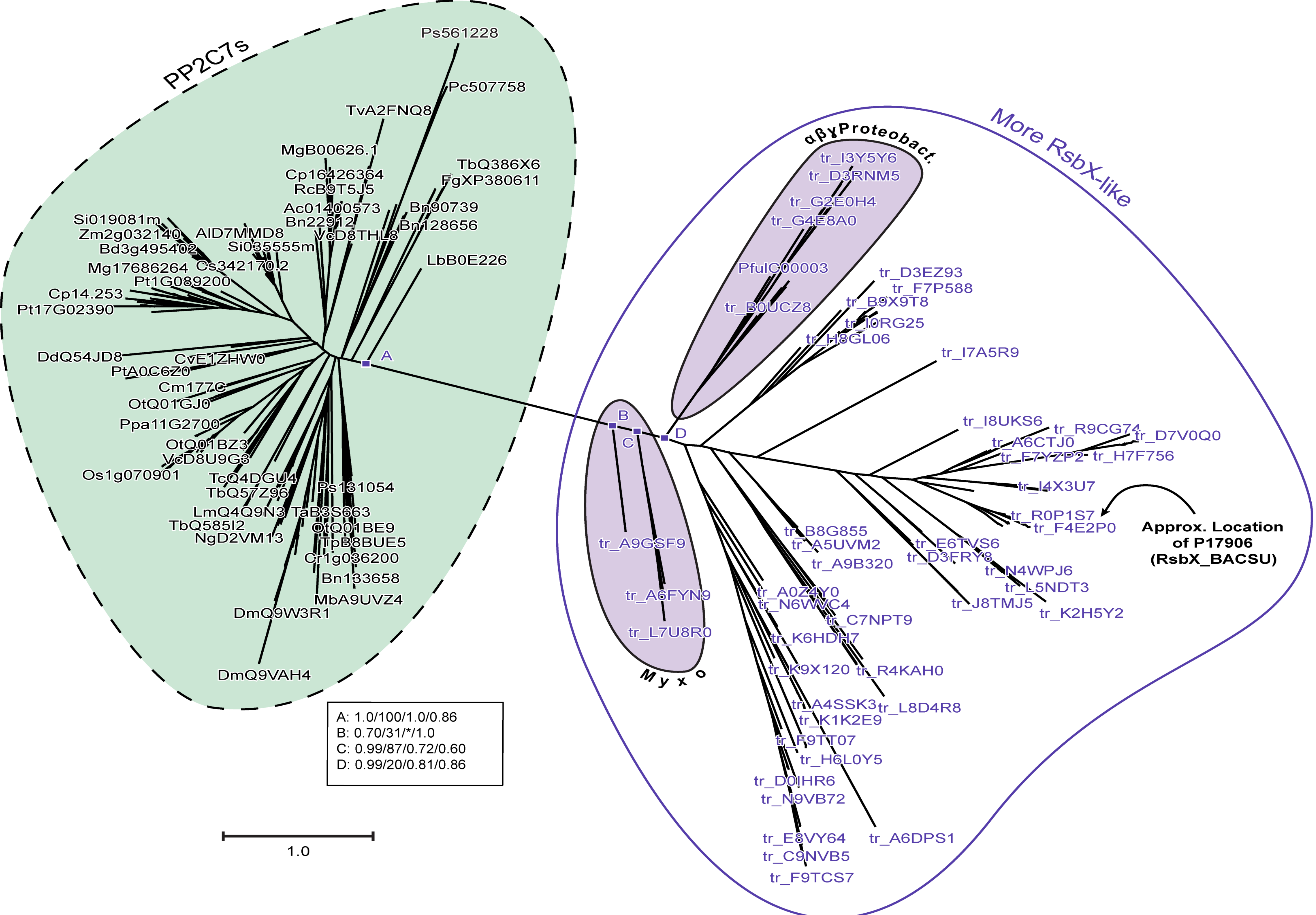

Fig E: Phylogenetic tree of PP2C7s with "More RsbX-Like" bacterial "GN" Group II PP2C sequences. The PP2C7 set is a very large and diverse one (238 sequences) and the bacterial Group II sequences are of the "GN" type, from the "More RsbX-Like" assemblage (149 sequences) (sequence varieties described in the text). Unrooted phylogenetic tree inference was performed as outlined in "Materials and Methods." The most crucial nodes are labeled. Node support values with the four inference methods (PhyML [aBayes], RAxML [RBS], MrBayes [PP], and PhyloBayes_MPI [PP]) are tabulated in the Figure, separated by slashes ("/").Support values for all trees are summarized in Table N in S1 File. The cluster of sequences from αβγ-Proteobacteria is indicated. "Myxo" designates sequences from Myxococcales (δ-proteobacteria). The approximate location in the tree of the reference sequence BsP17906 (RsbX_BACSU) is indicated. This tree is based on the amino acid sequence alignment presented in Fig. A in S2 file (Panel 5).
* = Single Myxococcales sequence unresolved in this tree.

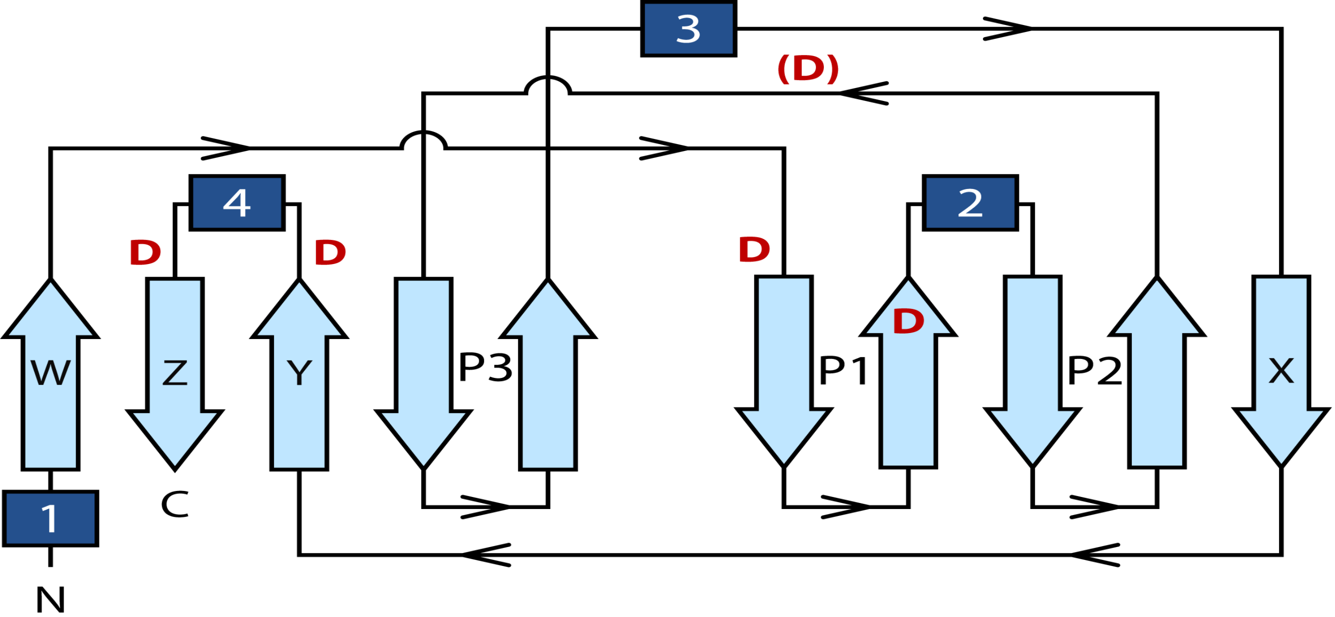

Fig F: Secondary structure diagram of solved PP2C structures. Diagrams of secondary structures of solved PP2C proteins were accessed as detailed in "Materials and Methods". This representation shows beta-strand elements organized around three sets of hairpin beta turns (designated "P1", "P2", and "P3" [i.e. first pair, second pair, etc]). Individual beta strands (designed "W", "X", "Y", "Z") are labeled from the amino terminus ("N") to the carboxy terminus ("C"). In combination these sets of beta strands form a beta sheet. The locations of universally conserved metal-coordinating aspartate residues ("D") are noted. These correspond to classic sequence Motifs 1 and 2 [84] (within the P1 beta strand pair), and 8 and 11 (in the loop between opposing beta strands Y and Z). The aspartate in parentheses "(D)" represents the residue in classic Motif 5, which is not conserved in all PP2C sequences. Boxed regions (numbered consecutively from the amino terminus) represent regions with more variable composition of secondary structure elements, but always including alpha helices which encase the central beta sheet in an alpha-beta sandwich. See Fig. 7 for a depiction of a structure-guided sequence alignment where the classic sequence motifs are shown in combination with conserved secondary structure elements, and metal-coordinating aspartate residues (D) are depicted. Differences between the aspartate composition of different PP2C sequence groups and the proposed evolutionary explanation are discussed in the text.

Fig G: Large Reference PP2C7 Alignment

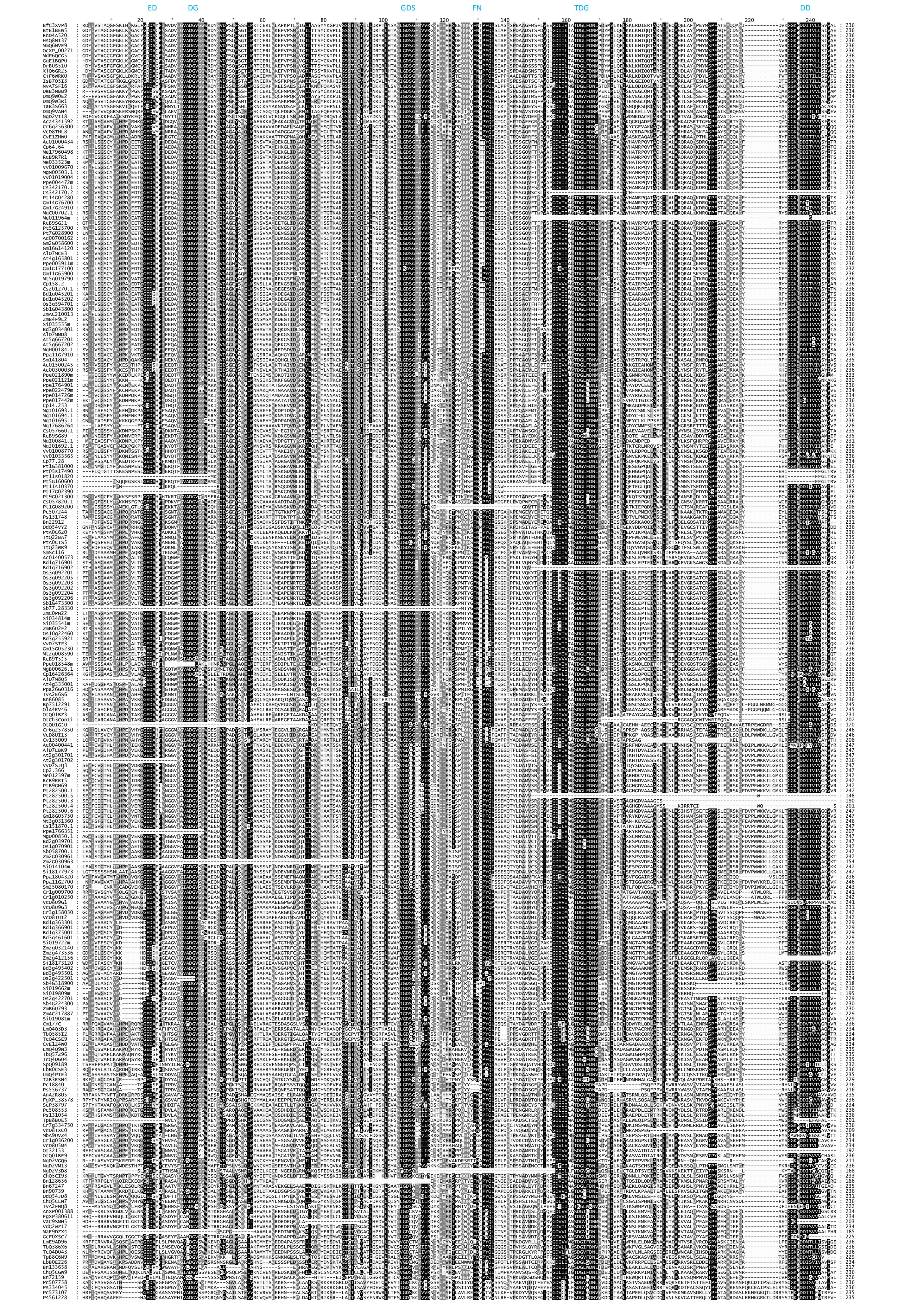

Fig G: Large reference PP2C7 alignment. Candidate PP2C7 sequences were retrieved, validated, and aligned as detailed in "Materials and Methods". See Table A in S1 File for a list of PP2C7 sequences. Above the alignment, in blue, are presented consensus residues forming landmark portions of classic Motif 1 ("ED"), Motif 2 ("DG"), Motif 5 ("GDS"), Motif 8 ("TDG"), and Motif 11 ("DD"). A signature characteristic of PP2C7s, and nearly invariant, is the presence of "FN" or "FD" in Motif 6 (also given in blue). A simplified secondary structure diagram of solved PP2C structures is given in Fig F in S2 File. A structure-guided alignment of PP2C7 sequences together with bacterial Group II, eukaryotic PP2C and bacterial Group I PP2C sequences is given in Fig. 7.
